# Unraveling the origin of Cladocera by identifying heterochrony in the developmental sequences of Branchiopoda

**DOI:** 10.1186/1742-9994-10-35

**Published:** 2013-06-19

**Authors:** Martin Fritsch, Olaf RP Bininda-Emonds, Stefan Richter

**Affiliations:** 1 Lehrstuhl für Allgemeine und Spezielle Zoologie, University of Rostock, Universitaetsplatz 2, 18055 Rostock, Germany; 2 Fakultät V, Institut für Biologie und Umweltwissenschaften (IBU), AG Systematik und Evolutionsbiologie, Carl von Ossietzky Universität Oldenburg, Carl von Ossietzky Str. 9-11, 26111, Oldenburg, Germany

**Keywords:** Developmental Sequences, Heterochrony, Nervous System Development, Parsimov Event-pairing, Progenesis of Cladocera

## Abstract

**Introduction:**

One of the most interesting riddles within crustaceans is the origin of Cladocera (water fleas). Cladocerans are morphologically diverse and in terms of size and body segmentation differ considerably from other branchiopod taxa (Anostraca, Notostraca, Laevicaudata, Spinicaudata and Cyclestherida). In 1876, the famous zoologist Carl Claus proposed with regard to their origin that cladocerans might have evolved from a precociously maturing larva of a clam shrimp-like ancestor which was able to reproduce at this early stage of development. In order to shed light on this shift in organogenesis and to identify (potential) changes in the chronology of development (heterochrony), we investigated the external and internal development of the ctenopod *Penilia avirostris* and compared it to development in representatives of Anostraca, Notostraca, Laevicaudata, Spinicaudata and Cyclestherida. The development of the nervous system was investigated using immunohistochemical labeling and confocal microscopy. External morphological development was followed using a scanning electron microscope and confocal microscopy to detect the autofluorescence of the external cuticle.

**Results:**

In Anostraca, Notostraca, Laevicaudata and Spinicaudata development is indirect and a free-swimming nauplius hatches from resting eggs. In contrast, development in Cyclestherida and Cladocera, in which non-swimming embryo-like larvae hatch from subitaneous eggs (without a resting phase) is defined herein as pseudo-direct and differs considerably from that of the other groups. Both external and internal development in Anostraca, Notostraca, Laevicaudata and Spinicaudata is directed from anterior to posterior, whereas in Cyclestherida and Cladocera differentiation is more synchronous.

**Conclusions:**

In this study, developmental sequences from representatives of all branchiopod taxa are compared and analyzed using a Parsimov event-pairing approach. The analysis reveals clear evolutionary transformations towards Cladocera and the node of Cladoceromorpha which correspond to distinct heterochronous signals and indicate that the evolution of Cladocera was a stepwise process. A switch from a strategy of indirect development to one of pseudo-direct development was followed by a shift in a number of morphological events to an earlier point in ontogenesis and simultaneously by a reduction in the number of pre-metamorphosis molts. A compression of the larval phase as well as a shortening of the juvenile phase finally leads to a precocious maturation and is considered as a gradual progenetic process.

## Introduction

Cladocera (water fleas) constitute the morphologically most diverse taxon of Branchiopoda [1] and might have been in existence since the Permian era [2-5]. Water fleas are distributed circumglobally and mostly occur in temporary or permanent freshwater pools, though a few species have colonized brackish or marine habitats [2,3,6]. The origin of the relatively small water fleas, as opposed to the 'large branchiopods' (Anostraca, Notostraca, Laevicaudata, Spinicaudata and Cyclestherida), is still one of the most interesting riddles in crustacean evolution. One explanation was put forward by Carl Claus in 1876, who claimed that cladocerans had evolved from free-swimming 'conchostracan' larvae (clam shrimps).

When adult cladocerans (particularly Ctenopoda) are compared with clam shrimp larvae (e.g. Spinicaudata), several striking similarities are indeed observable. The bivalved carapace covers six phyllopodous trunk limbs, the head and the posterior end of the body remain free, and the antenna is the main swimming organ. The evolutionary process suggested by Claus has since been called progenesis, although the term neoteny has regularly been used to describe it too [2,7-12]. Progenesis [13] is where the somatic development of a larva or juvenile is stopped by the onset of premature sexual maturation [14,15]. Neoteny [16], on the other hand, sees somatic development delayed, with the result that larval or juvenile characters can be still present in adults. Both processes result into paedomorphosis (retention of larval or juvenile morphology in adults, [17-19]). In the case of cladocerans this would mean that the ancestor of the water fleas was a 'precocious metanauplius larva which becomes mature without growing up' [8: 130], or in other words that cladocerans evolved from a free-swimming sexually mature larva of a conchostracan-like ancestor.

If the evolution of Cladocera was progenetic, the ancestor would have looked as described above: six limb pairs, the head and the posterior end remaining free and the antenna used mainly for swimming. All these features are recognizable in the last larval stage (which is also known as the Heilophora larva [4,20,21]) and in early juvenile stages of some spinicaudatans (see below)).

Strictly speaking, however, this theory on the origin of Cladocera is still under debate. Many researchers have rejected the notion that Cladocera could have a common origin and argued that the four cladoceran lineages (Anomopoda, Ctenopoda, Onychopoda and Haplopoda) evolved independently e.g. [7,12,22-26]. All recent morphological, molecular, and combined morphological and molecular studies, on the other hand, have supported the theory that Cladocera is monophyletic [6,26-32]. The sister group to Cladocera is Cyclestherida, which is not monotypic, as generally believed, but consists of a number of species within *Cyclestheria*[33]. Cladocera and Cyclestherida together form Cladoceromorpha [6,31,32,34,35], whose sister group is Spinicaudata *sensu* Olesen [29,30]. On the basis of morphological data, the sister group of Cladoceromorpha + Spinicaudata (=Onychocaudata *sensu* Olesen and Richter [36]) is Laevicaudata (another clam shrimp taxon [29,30]), though some molecular data favor a sister group relationship between Notostraca (tadpole shrimps) and Onychocaudata, with Laevicaudata constituting the sister group to all other phyllopods [6,32,35]. Recent metazoan phylogenies based on protein-coding genes and EST data [37,38]), however, support the monophyly of the Diplostraca *sensu* Olesen and Richter [36] and the sister group relationship between Laevicaudata and Onychocaudata.

The phylogeny of recent Branchiopoda *sensu* Olesen [29,30], then, appears to be settled: (Anostraca (Notostraca (Laevicaudata, (Spinicaudata (Cyclestherida and Cladocera))))).

Various reproductive strategies are found within branchiopods (gonochorism, parthenogenesis, cyclic parthenogenesis, hermaphroditism and androdioecy [4]), and two kinds of eggs – subitaneous or resting – are produced depending on the strategy. Resting eggs are almost exclusively gamogenetic [1,4,39,40] and represent a 'highly specialized adaption to colonize extreme environments' [41] and a means of surviving harsh environmental conditions [42]. Free-swimming nauplius larvae generally hatch from gamogenetic resting eggs but are also reported to emerge from parthenogenetic subitaneous eggs in some anostracans (ovoviviparity e.g. [1,4,43,44]), notostracans [1,45] and spinicaudatans [46-48]. The parthenogenetic reproduction of some Anostraca, Notostraca and Spinicaudata is considered to be an additional, secondary developmental strategy [4,41,45,49].

Hatchlings or nauplius larvae from the gamogenetic resting eggs of anostracans, notostracans, laevicaudatans and spinicaudatans possess a uniramous antennula, a biramous antenna and a three-segmented mandibular palp (orthonauplius larva); additional segments and appendages develop over the course of successive larval stages [50]. This developmental strategy is termed anamorphic indirect development after [51-53] and is also reported for the Cambrian fossil *Rehbachiella kinekullensis* Müller 1983 and the Devonian fossil *Lepidocaris rhyniensis* Scourfield 1926 [54-58]. In view of the strongly supported monophyly of Branchiopoda and the evidence of the fossil record, anamorphic indirect development can be said to represent the plesiomorphic branchiopod developmental strategy.

Cladoceromorpha, on the other hand, have abandoned the typical anamorphic developmental strategy [22,44,59]. In Cladocera and Cyclestherida, a gamogenetic phase resulting in resting eggs which develop directly and a parthenogenetic phase resulting in subitaneous eggs which develop 'pseudo-directly' ('pseudo-direct development') (see below) alternate over a heterogonous life cycle. The parthenogenetically produced subitaneous eggs of cyclestheridans and cladocerans develop within the dorsal brood pouch under the carapace of the mother animal (Cyclestherida [60-62]; Cladocera [1,4]). Within them, various embryonic stages develop which are morphologically clearly distinguishable and separated by embryonic molting cycles (Cyclestherida [60-62]; Cladocera [49,50,63-65]). The non-swimming embryo-like larvae [49] which hatch are carried under the dorsal part of the carapace (Cyclestherida: [60-62]; Cladocera: e.g. [3,40,44,49]). These almost immobile embryo-like larvae possess several embryonic characteristics including yolk and undifferentiated segments and appendages (Cyclestherida [61]; Cladocera [3,49,64]) and have for this reason also been called embryonized larvae or embryos [6,29,61-66] (Cyclestherida [60-62,67]; Cladocera [1,3,4,39]). Additional segments and appendages differentiate over consecutive molting cycles. The end of this developmental phase is characterized by the process of metamorphosis (transformation into juveniles or post-larvae). The first juvenile stages are capable of swimming and resemble the adult animal in external morphology (Cyclestherida [67]; Cladocera [12,40,44]). Though this developmental strategy is often regarded as direct development after [51,52], under the accepted definition of direct development hatched juveniles possess their full complement of body segments and are similar in terms of morphology to adult animals. The hatched embryo-like larvae of Cyclestherida and Cladocera, however, are neither morphologically similar to the adults nor in possession of the full number of body segments. In Cladoceromorpha, then, the strategy of parthenogenetic reproduction involving subitaneous eggs and embryo-like larvae is actually a different kind of development which we term 'pseudo-direct'. It is a strategy which results in rapid population growth in newly colonized habitats. Extreme cases of rapid next generation growth have been observed in some anomopod, ctenopod and onychopod specimens, where almost fully developed embryo-like larvae start to produce parthenogenetic subitaneous eggs while they are still being carried in the dorsal brood pouch e.g. [39,40,64,68].

Gamogenetic reproduction involving resting eggs in Cyclestherida (where the eggs are enclosed in an ephippium [67]) and Cladocera is normally associated with a deterioration in environmental conditions (illumination, temperature, oxygen, food availability, crowding or a shortening of the photoperiod: Cyclestherida [67]; Cladocera [3,4,40,42]). The hatchlings or juveniles from gamogenetic resting eggs look like replicas of the adults (Cyclestherida [67]; Cladocera [12,40,44]). This strategy of apparently direct development appears to be even more derived from the ‘original’ strategy of anamorphic indirect development displayed by most large branchiopods. In Cladocera, the first generation to hatch from resting eggs is capable of rapid growth and maturation and exhibits a high rate of parthenogenetic reproduction, while sexual reproduction is repressed [42].

In our view, such a heterogonic life cycle was already present in the ground pattern of Cladoceromorpha, where a gamogenetic phase resulting in resting eggs with direct development alternated with and a parthenogenetic phase resulting in subitaneous eggs with pseudo-direct development. However, two findings might challenge this conclusion.

Botnariuc and Viña Bayés (1977) documented a free-swimming Heilophora larva seemingly belonging to *Cyclestheria hislopi* that they believed hatched as a nauplius larva from resting eggs [20]. The Heilophora larvae in question were collected in temporary water pools in Cuba and documented only once. No other studies on the development of *C*. *hislopi*[62,67] have ever described a free-swimming Heilophora larva - on the contrary, they have all reported hatchlings from resting eggs which emerged in possession of the adult morphology. It therefore appears possible that the authors confused the larva in question with a spinicaudatan larva resembling *Eulimnadia braueriana*. *E*. *braueriana* larvae correspond almost exactly in shape to the schematic drawing of the supposed Heilophora larva presented by Botnariuc and Viña Bayés [20]. In addition, both the Heilophora larva in question and those of *E*. *braueriana* exhibit a single median posteriorly directed labral spine (*E*. *braueriana* see Figure nine a and ten A in [66]) that is not present in any stage of cyclestheridan development, either in the pseudo-direct or in the direct phase [60,61,67].

Another challenge to our hypothesis is the fact that the cladoceran *Leptodora kindtii* (Haplopoda) actually pursues a different developmental strategy, alternating subitaneous eggs with pseudo-direct development and resting eggs that hatch into free-swimming metanauplius larvae. This larva is very unique. Nowhere else in Branchiopoda do freshly-hatched free-swimming metanauplius larvae in possession of several postnaupliar segments and appendages occur. The presence of free-swimming metanauplius larvae in *L*. *kindtii*[3,7,21,39,44,49,64,69-73] might therefore be regarded as a secondary developmental strategy of the haplopod cladoceran lineage.

Could *C*. *hislopi* and *L*. *kindtii* have retained two different developmental strategies from the ground pattern of Cladoceromorpha? If we take the report of the free swimming *Cyclestheria* larva as fact, we have to accept that it hatched as an orthonauplius larva equipped with only three pairs of appendages. The hatching larva in the haplopods, however, is a metanauplius additionally equipped with six postnaupliar appendage pairs [69,70] - a unique situation in branchiopods. As the two differ significantly from each other, the argument for their common origin is weak. Moreover, a free swimming larva must have then been reduced at least three times in Anomopoda, Ctenopoda and Onychopoda as well as in most *Cyclestheria* populations. This is certainly not a parsimonious solution.

All this together, however, means that the original theory that cladocerans evolved by progenesis from a precocious conchostracan larva-like ancestor cannot be true [30,61] because free-swimming Heilophora-like larvae most probably no longer existed in the ancestral lineage of Cladocera. The only larva to appear in the ancestral lineage of Cladoceromorpha is a non-swimming embryo-like larva, which occurs as part of a strategy of pseudo-direct development. Nevertheless, the small size of cladocerans compared to both adult large branchiopods and, more importantly, their larvae still indicates some kind of heterochronic process obviously much more complicated than Claus believed.

To analyze this process and reconstruct the evolutionary transformation from a conchostracan-like ancestor to Cladoceromorpha and Cladocera, we investigated the pseudo-direct development of the marine ctenopod *Penilia avirostris* Dana 1849. Though *P*. *avirostris* is ecologically highly specialized and evolved from freshwater ctenopods which migrated through a favorable river estuary [40,74,75], it possesses characteristic ctenopod traits such as six pairs of phyllopodous filter-feeding trunk limbs [75] and a bivalved carapace and so we consider it here to be a good representative of Ctenopoda. Furthermore, recent phylogenetic analyses have shown that Ctenopoda and Anomopoda can be considered plesiomorphic in many characters e.g. [6,27,29,30], whereas Onychopoda and Haplopoda (Gymnomera) are considered to be more derived, exhibiting either six or four pairs of stenopodous limbs which evolved from phyllopodous limbs, and a carapace which is reduced to a brood pouch e.g. [3,6,27,29,30,39,76].

To document the chronology of development on the basis of internal and external morphology, we analyzed the progression of the nervous system and the first expression pattern of the neurotransmitter serotonin (5 Hydroxytryptophan), and used the autofluorescence effect [77,78] in combination with a confocal microscope to trace the growth of the cuticle. To pinpoint potential heterochronies we compared the development of *P*. *avirostris* with the development of representatives of all other branchiopod taxa. Because of the different developmental strategies and varying number of developmental stages exhibited by the specimens, a direct stage-by-stage comparison was not possible so we resorted to a 'Parsimov event-pairing approach’ to examine potential differences in the chronology of development.

## Results

### Pseudo-direct development in *Penilia avirostris*

In *P*. *avirostris*, fertilized eggs, embryos and embryo-like larvae are carried in the dorsal brood pouch under the carapace of the mother animal. Embryonic stages E I-IV are enclosed in the first (egg) membrane. Stage V hatches from the first egg membrane. Stages V and VI continue to grow and develop in the dorsal brood pouch and possess both embryonic (yolk) and naupliar larval characters (naupliar appendages, albeit not fully developed). They are thus considered embryo-like larvae (LV - VI). The staging in *P*. *avirostris* is in accordance with the development of the ctenopods *Sida cristallina* and *Diaphanosoma brachyurum*[64].

**Figure 1:**
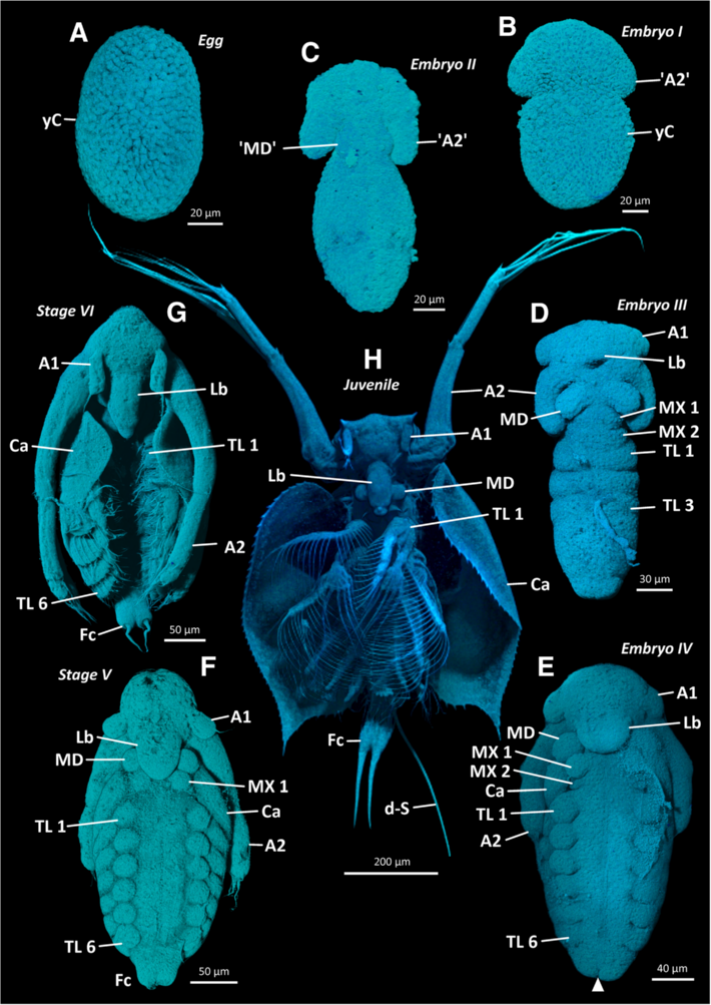
***Penilia avirostris*, autofluorescence images of embryonic**, **embryo-like larval and juvenile stages (A-H) of the external morphology (ventral view).** A1 antennula, A2 antenna, Ca carapace, d-S dorsal setae, Fc furca, Lb labrum, MD mandible, MX 1/2 maxillulae and maxilla, TL thoracic limbs, yC yolk cell(s).

The eggs (Figure 1A) are ellipsoid in shape, about 110 μm long and possess a high number of yolk cells.

In E I (Figure 1B), the yolk cells are distributed throughout the whole of the developing embryo, length is about 150 μm and the anterior pole starts to differentiate. Laterally on both sides, the developing antenna ('A2') (Figure 1B) is present as a small bulge. No differentiation is seen in the posterior region of the embryo.

E II (Figure 1C) is about 160 μm long and the anterior region is further differentiated. The antenna (Figure 1C) is elongated posteriorly and, distally, the first signs of the differentiating exo- and endopodite (not shown) are distinguishable. Medially, the mandibular knobs ('MD') (Figure 1C) begin to form. The post-mandibular region is still undifferentiated.

**Figure 2:**
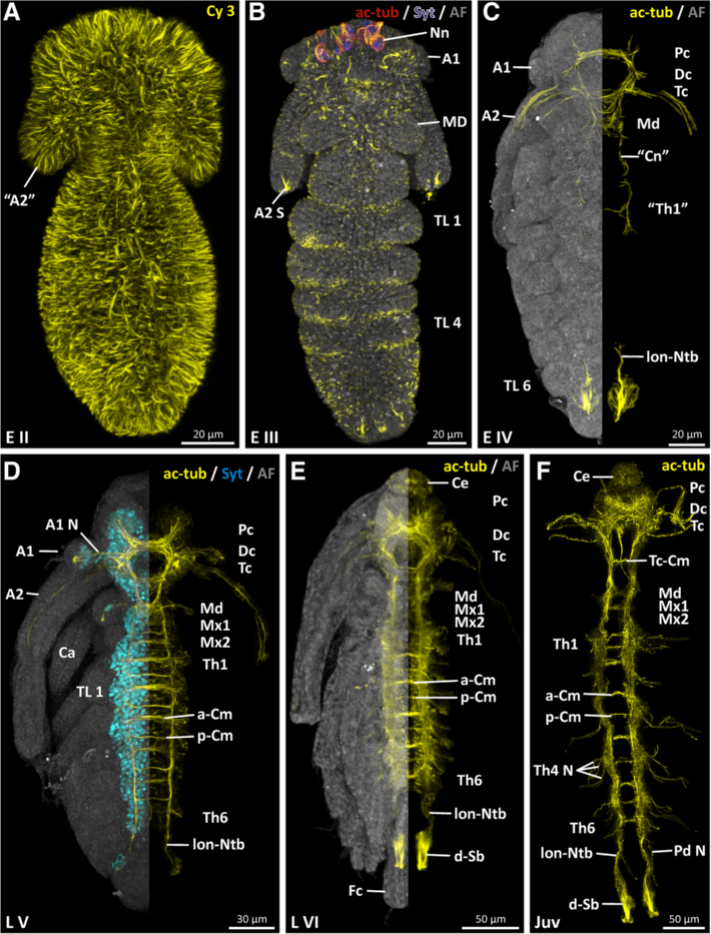
***Penilia avirostris, *embryonic, embryo-like larval and juvenile stage nervous system (ventral view). A** unspecific signal (yellow) of the coupled fluorochrome of the secondary antibody. **B** autofluorescence (grey) and primary neurons (red and blue) at the anterior end of the embryo. **C**, **E** combined autofluorescence (grey) image and nervous system (yellow) image. **D** combined autofluorescence (grey), nuclei distribution pattern (cyan) and nervous system (yellow) image. **F** nervous system (yellow). A1 antennula, A1 N antennula nerve, A2 antenna, A2 S antennula seta, ac-tub acetylated tubulin, AF autofluorescence, a-Cm anterior commissure, Ca carapace, Ce compound eye, Cn connective, Dc deutocerebrum, d-Sb dorsal setae basis, Fc furca, lon-Ntb longitudinal neurite bundle, MD mandible, Md mandibular neuromere, Mx1/2 maxillula/maxilla neuromere, Nn neuron(s), Pc protocerebrum, Pd N proctodaeum nerve, p-Cm posterior commissure, Syt Sytox, Tc tritocerebrum, Tc-Cm tritocerebral commissure, Th thoracic neuromere, TL thoracic limbs.

E III (Figure 1D) is about 230 μm long. Laterally, on both sides of the head, the antennulae (A1) are present as little knobs (Figure 1D); medially the labrum (Lb) anlage (Figure 1D) protrudes ventrally, the antenna (Figure 1D) is more elongated and the mandibular knobs are larger (Figure 1D). At the tip of the developing exo- and endopodite (not labeled) of the antenna, the first short setae (A2 S) can be distinguished (Figure 2B). In the post-mandibular region the knobs of the maxillula and maxilla (MX 1/2) are visible (Figure 1D). In the trunk region, the first three to four thoracic limb (TL) anlagen (1–3, 4: Figures 1D, 2B) are identifiable.

The last embryonic stage (E IV) is about 300 μm long (Figure 1E). The head appendages are more differentiated and the antenna (Figure 1E) extends posteriorly to the second thoracic segment. In comparison to the maxillula, the maxilla remains smaller in size (Figure 1E). The carapace (Ca) anlage appears dorsally in the region of the maxillula and maxilla segment and protrudes ventrally on both sides, between the antenna and the maxillula (Figure 1E). In the trunk region, six thoracic limb anlagen (Figure 1E), including distinguishable podomeres, are present. At the posterior end, a dorso-ventral furrow (arrowhead) appears (Figure 1E), creating two lobes, the future furcal rami.

The first embryo-like-larval stage (L V) is about 340 μm long (Figure 1F). The head is more elongated; the antenna (Figure 1F) extends posteriorly to the fourth thoracic segment. All developing appendages and their podomeres are equipped with several setae, except the uniramous antennula (Figure 1F), which possesses one long seta only. The knob of the maxillula (Figure 1F) is well discernible directly posterior to the mandible, but no external maxilla is present. The carapace (Figure 1F) extends to the first thoracic segment. At the posterior end of the trunk, two developing furcal rami (Fc) are present (Figure 1F).

In L VI, the embryo-like larva is about 420 μm in length and considerably more developed in terms of external morphology (Figure 1G). The anterior trunk limbs (Figure 1G) are well differentiated and exhibit a typical comb-like shape. The antennula (Figure 1G), still equipped with only one seta, is longer, as is the labrum (Figure 1G). The antenna (Figure 1G) extends to the sixth thoracic segment. The carapace (Figure 1G) starts to envelope the embryo-like larva laterally and covers the first two to three trunk limbs.

The first juveniles in *P*. *avirostris* are about 700 μm long (Figure 1H). The most obvious difference to the last embryo-like-larval stage is that the bivalve carapace (Figure 1H) envelopes the whole trunk, but not the head region and the posterior end of the body. The antenna (Figure 1H), the main swimming organ, is mostly carried in an upright position. The antennula (Figure 1H) is significantly longer than in the previous stage and bears now short setae (not labeled), the aesthetascs, in addition to the long seta. The podomeres of the trunk limbs (Figure 1H) possess long setae forming the filter feeding apparatus. Dorsally at the posterior end, two long setae (d-S) are present and the furca is equipped with two furcal rami which extend posteriorly (Figure 1H).

### Development of the nervous system in *Penilia avirostris*

Nervous system tissues are not detectable in either E I or II (E II: Figure 2A). In E III, the first signs of the nervous system appear in the anterior head region (Figure 2B) with the differentiation of the first neurons (Nn) (Figure 2A). In contrast to the other cells spread over the embryo, these neurons possess a relatively large nucleus. No additional neuronal structures can be identified in E III.

**Figure 3:**
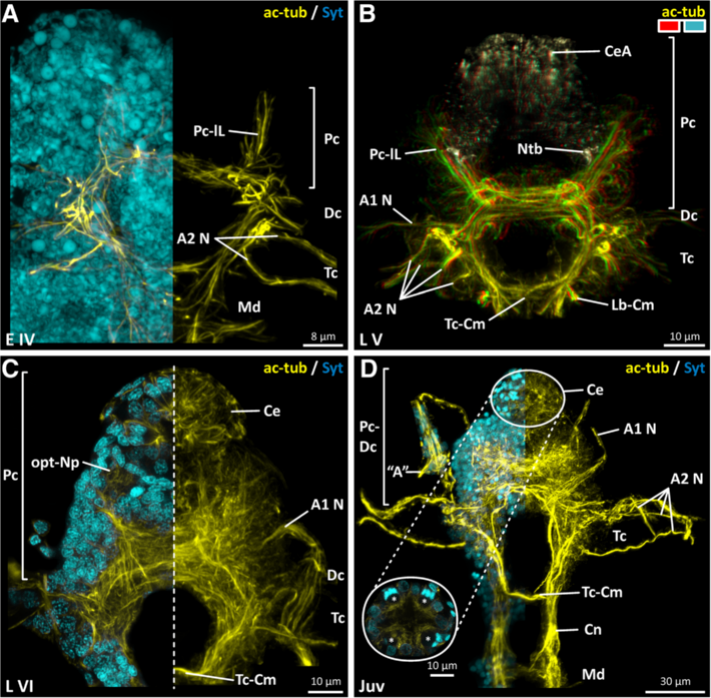
***Penilia avirostris, *development of the visual system and the brain architecture (ventral view). A**, **C** combined nuclei (cyan) and nervous system (yellow) image. **B** dorsally the compound eye anlagen (beige) and the remaining brain (yellow) depicted in 3D. **D** combined nuclei (cyan) and nervous system (yellow) image including a higher magnified image of a horizontal section of the compound eye. A antennula aesthetasc(s), A1 N antennula nerve, A2 N antenna nerve(s), ac-tub acetylated tubulin, Ce compound eye, CeA compound eye anlagen, Cn connective, Dc deutocerebrum, Lb-Cm labral commissure, Md mandibular neuromere, Ntb neurite bundle, opt-Np optical neuropil, Pc protocerebrum, Pc-lL protocerebral lateral lobes, Syt Sytox, Tc tritocerebrum, Tc-Cm tritocerebral commissure, asterisk ommatidia.

In E IV, the head encloses the anlagen of a naupliar nervous system: proto-, deuto- and tritocerebrum (Pc, Dc, Tc) and a pair of primordial mandibular ganglia (Md) (Figure 2C). In the protocerebrum, the first neurites of the developing lateral lobes (Pc-lL) are present (Figure 3A), while from the deuto- and tritocerebrum first nerves of the developing antennula and antenna (A2 N) extend laterally (Figure 3A). Postnaupliarly (posterior to the mandibular region), two slender longitudinal neurite bundles, the future connectives ('Cn'), extend posteriorly into the trunk region (Figure 2C). From the posterior end, two slender longitudinal neurite bundles (lon-Ntb) extend anteriorly (Figure 2C). A connection to the anterior nervous system is not yet present.

**Figure 4:**
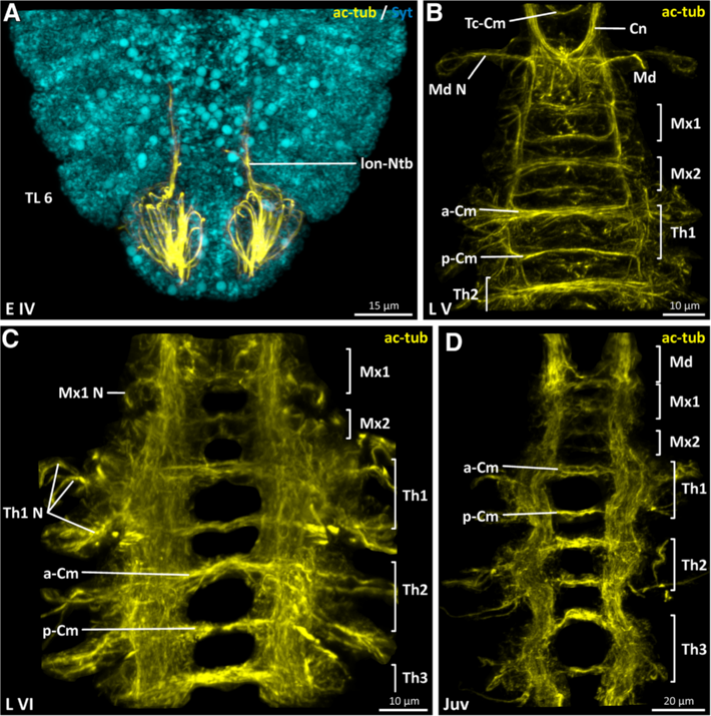
***Penilia avirostris, *A combined nuclei (cyan) and nervous system (yellow) image shows the innervation of the posterior end of the body, B-D nervous system data illustrating the development of the subesophageal region (ventral view).** ac-tub acetylated tubulin, a-Cm anterior commissure, Cn connective, Md mandibular neuromere, Md N mandibular neuromere nerve, Mx1/2 maxillula/maxilla neuromere, Mx1 N maxillula neuromere nerve, Ntb neurite bundle, opt-Np optical neuropil, p-Cm posterior commissure, Pc-lL protocerebral lateral lobes, Syt Sytox, Thoracic neuromere(s), Th N thoracic neuromere nerve(s), Tc-Cm tritocerebral commissure.

In L V, the nervous system is considerably more advanced (Figure 2D). The ventral nerve cord in particular is distinctly more developed than in the previous stage. In the head region, the architecture of the brain (proto-, deuto- and tritocerebrum) is intricately cross-linked (Figure 3B). The lateral lobes (Figure 3B) in the protocerebrum are more developed and expand antero-laterally on each side. On the antero-dorsal side, the confluent compound eye anlage (CeA), which starts to develop (Figure 3B), is connected dorsally by two lateral slender neurite bundles (Ntb) with the remaining protocerebral scaffold (Figure 3B). The deutocerebrum (Figures 2D, 3B) is only identifiable by the nerve of the antennula (Figure 3B) and is, anteriorly, directly connected to the protocerebrum (Figures 2D, 3B). The tritocerebral ganglia (Figures 2D, 3B) are directly connected with the posterior parts of the deutocerebrum. Posterior to the esophagus, the tritocerebral commissure (Tc-Cm) connects the tritocerebral ganglia transversally and forms the posterior arch of the circumoral brain (Figure 3B). Ventrally, a tritocerebral labral commissure (Lb-Cm) is present (Figure 3B). Laterally, each tritocerebral ganglion possesses four developing nerves (Figure 3B) which extend into the antenna. In the mandibular neuromere (Figure 2D) one lateral nerve (Md N) enters the mandibular appendage on each side (Figure 4B). Postnaupliarly, in the maxillular, maxillar (Mx1/2) and each of the six thoracic segments (Th) (Figures 2D, 4B), a pair of primordial ganglia is present, longitudinally connected by two parallel connectives (Figure 4B). A relatively wide anterior and more slender posterior commissure (ant/pos-Cm) is present in each of the maxillular, maxillar and the first five thoracic neuromeres (Figure 4B). The sixth thoracic neuromere (Figure 2D) only exhibits a slender anterior commissure. Laterally, on each primordial thoracic ganglion, the developing nerves start to extend into the periphery of the appendage. Posterior to the sixth thoracic neuromere, two slender longitudinal neurite bundles (lon-Ntb) are connected with the telsonic region (Figure 2D).

In L VI, the nervous system is well developed. All parts of the brain are robustly connected to each other. Within the protocerebral scaffold (Figure 3C) distinct neuropilar subdivisions are distinguishable. Dorsally a well differentiated fused compound eye (Ce) is present (Figures 2E, 3C). The ommatidial neurites converge into two optical tracts (not shown) which enter a single optical neuropil (opt-Np) on each side of the protocerebrum antero-dorsally (Figure 3C). The division of the optical neuropils into a visual tectum and medulla is not discernible using the applied staining methods. Within the fused compound eye, ten ommatidia are present. Subesophageally, in the maxillular, maxillar and the six segments of the trunk region, a pair of well developed ganglia is identifiable (Figure 2E). Each maxillular ganglion sends one nerve (Mx1 N) laterally into the maxillula (Figure 4C). No lateral nerves are detectable in the maxillar neuromere (Figure 4C). The pair of thoracic ganglia in the sixth segment is transversally connected, as are all the other trunk neuromeres, by a relatively wide anterior and more slender posterior commissure (Figure 2E). Laterally, from each thoracic ganglion three nerves (e.g. Th1 N) (Figure 4C) extend into each trunk limb.

In the juvenile, the whole nervous system is more advanced and more longitudinally stretched than in the previous stage (Figure 2F). The architecture of the brain (proto-, deuto- and tritocerebrum) is intricately cross-linked, exhibiting several neuropilar sublayers (Figure 3D). A nauplius eye complex is not discernible using these staining methods. At the tip of the antennula, beneath the short aesthetascs, a small set of bipolar neurons (not labeled) is detectable, one neurite from which projects into the aesthetascs and one branch into the antennula nerve (Figure 3D). The ventral nerve cord exhibits a high degree of differentiation. In comparison, the maxillular neuromere has decreased in size (Figure 4D). All the thoracic ganglia send three well developed nerves (e.g. Th4 N) laterally into the trunk appendages (Figure 2F). At the posterior end of the body, the two longitudinal neurite bundles which extend posteriorly send one nerve branch (Pd N) on each side to innervate the proctodaeum (Figure 2F), and another (not labeled) to innervate the dorsal seta (d-Sb) (Figure 2F).

### Serotonin-like immunoreactivity in *Penilia avirostris*

**Figure 5:**
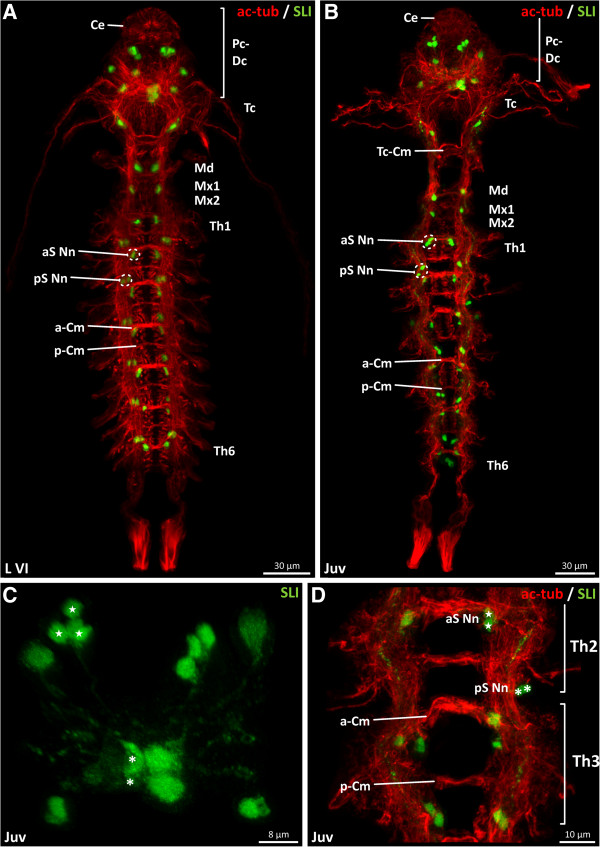
***Penilia avirostris, *last embryo-like larval and juvenile stage, serotonin-like immunoreactive expression pattern (ventral view). A**, **B** overview of the nervous system (red) and its serotonin-like expression pattern (green). **C** protocerebral serotonin-like immunoreactivity. **D** serotonin-like immunoreactivity (green) in the thoracic neuromeres (red) two and three. aS-Nn anterior serotonin-like neuron(s), a-Cm anterior commissure, Ce compound eye, Dc deutocerebrum, Md mandibular neuromere, Mx1/2 maxillula and maxilla neuromere, p-Cm posterior commissure, Pc protocerebrum, pS-Nn posterior serotonin-like neuron(s), SLI serotonin like immunoreactivity, Tc tritocerebrum, Th1-6 thoracic neuromere.

Serotonin-like immunoreactivity (SLI) in *P*. *avirostris* is detectable for the first time in L VI (Figure 5A). In the protocerebrum (shown in the first juvenile: Figure 5C), several serotonin-like immunoreactive (SL-ir) neurons are present. Ventrally, four monopolar neurons (asterisks indicate two of them), showing distinct SLI project their neurites into the median protocerebral neuropil (Figure 5C). On the dorsal side of the protocerebral scaffold, directly beneath the fused compound eye, a pair of three lateral SL-ir somata (stars indicate three of them) is present (Figure 5C). Four more serotonin-positive neurons, two on each side, are identifiable in the protocerebral scaffold. In each tritocerebral ganglion, one SL-ir soma is detectable (Figure 5A). In the mandibular and maxillular neuromere, two highly serotonin-like immunoreactive neurons are identifiable accordingly. In trunk segments one to five, a set of four anterior SL-ir somata and four posterior SL-ir somata is present. The sixth thoracic neuromere possesses only four anterior SL-ir neurons.

In the first juvenile stage, the serotonin expression pattern is more distinct than in previous stages (Figure 5B). All serotonin-positive somata exhibit a high level of immunoreactivity. In the ventral nerve cord, two anterior pairs of two SL-ir somata (aS Nn) are located near the anterior commissure in each thoracic neuromere (Figure 5D), and two pairs of posterior SL-ir somata (pS Nn) are located more posteriorly and laterally in each thoracic segment (Figure 5D).

## Discussion

The study of cladoceran development has a long tradition, but most researchers have focused on the external development of Anomopoda e.g. [44,63,79,80], Ctenopoda e.g. [64,81-84], Onychopoda e.g. [40,68] and Haplopoda e.g. [49,71,72,85-87]. This developmental study into *Penilia avirostris* constitutes the first attempt to describe both external and internal morphology in parallel in a cladoceran species. Though a substantial number of papers have discussed and compared external larval development e.g. [6,29,30,61,62,88-91] and nervous system development e.g. [87,92-95] in various branchiopods, the focus of this study is not a review of existing literature on the development of cladocerans and other branchiopod taxa, its aim is to present a practical and reproducible approach to investigating and envisioning a potential evolutionary transformation based on the example of the origin of Cladocera. To analyze the potential transformation in question, we included representative data from our earlier studies on the development of the notostracan *Triops cancriformis*[95], the laevicaudatan *Lynceus biformis*[78], the spinicaudatan *Leptestheria dahalacensis*[60] and the cyclestheridan C*yclestheria hislopi*[60]. To obtain a comprehensive set of external and internal developmental data for all recent branchiopod taxa, we also included developmental data pertaining to the anostracan *Branchinella* sp. (Frase and Richter, in preparation) in our analysis.

The developmental data pertaining to Anostraca, Notostraca, Laevicaudata and Spinicaudata are based on developing anamorphic larvae which hatched from resting eggs, while the data pertaining to Cyclestherida and Cladocera are based on the offspring which developed from parthenogenetic subitaneous eggs and are carried under the carapace (embryo-like larvae sensu Fritsch and Richter [60]). Though these developmental strategies differ considerably, they appear, as mentioned above, plesiomorphic for the respective branchiopod taxa.

Cladoceromorphan pseudo-direct development, which involves subitaneous eggs, hatching (i.e., the casting of the inner egg membrane as observed in Anomopoda and Ctenopoda: [44,64,96]) and embryo-like larvae, does seem to be more comparable with the anamorphic indirect developmental strategy in Anostraca, Notostraca, Laevicaudata and Spinicaudata than the gamogenetic reproduction and development in Cyclestherida and the Cladocera, where fully differentiated juveniles hatch from resting eggs [12,40,44,67].

### Parsimov event-pairing analysis: ancestral developmental sequences and event movements

**Figure 6:**
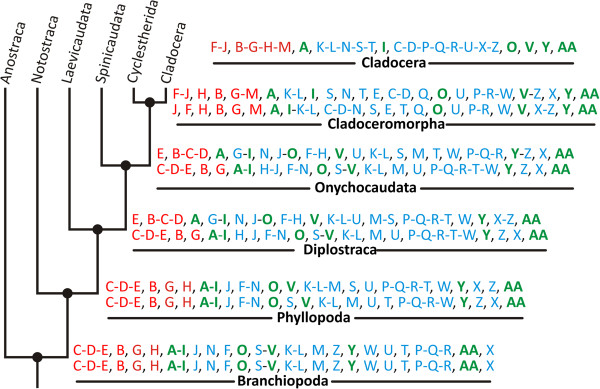
**Ancestral developmental sequences ACCTRAN (upper row) and DELTRAN (lower row) developmental sequences for the crown-group nodes of Branchiopoda, Phyllopoda, Diplostraca, Onychocaudata and Cladoceromorpha.** The developmental sequence obtained for *Penilia avirostris* (Cladocera) is additionally depicted for comparison. Red represents embryonic development and blue larval development. Green letters represent the hatching and molting processes. Note the last larval (metamorphosis) molt is transferred from 'AA' in the Onychocaudata to 'Y' in the Cladoceromorpha and to 'O' in the Cladocera.

The analysis resulted in ancestral developmental sequences for the nodes of crown-group Branchiopoda, Phyllopoda, Diplostraca, Onychocaudata and Cladoceromorpha (Figure 6), i.e. the developmental sequences of the respective groundpatterns. ACCTRAN (favouring earlier character transformations with later reversals, also known as fast transformation; [97]) and DELTRAN (favouring later, parallel changes, also known as slow transformation; [97]) optimizations of equally parsimonious alternative character transformations resulted in slightly different sequences. The embryonic phase within the developmental sequence is defined as all ‘morphological’ events (all events apart of the hatching process and the molts) present before hatching of the resting eggs or casting of the first egg membrane. The larval phase within the developmental sequence is defined as all morphological and molting events after hatching and before metamorphosis, regardless of whether or not the larvae are free-swimming or embryo-like. In the case of Diplostraca, Onychocaudata, Cladoceromorpha and Cladocera all morphological events defined herein are present before metamorphosis. The metamorphosis process (the last larval molt and transition into a juvenile) of Diplostraca, Onychocaudata and Cladoceromorpha represents the end of the larval phase and results in a juvenile in which the adult bivalved morphology is already evident (modified from Olesen [50]).

**Figure 7:**
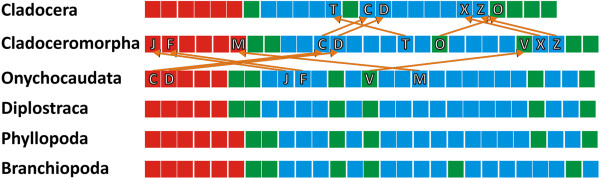
**Color coded DELTRAN developmental sequences for the crown-group nodes of Branchiopoda, Phyllopoda, Diplostraca, Onychocaudata and Cladoceromorpha and in addition the developmental sequence of Cladocera.** The color code illustrates the compression of events in the developmental phases, respectively and the shortening of the larval phase in Cladoceromorpha and Cladocera. Letters and arrows depicted, represent the result of the DELTRAN Parsimov event-pairing analysis from the node of Onychocaudata to Cladoceromorpha.

A comparison of the number and distribution of morphological events in the embryonic and larval phase and the number of larval molts is presented in Table 1 (Additional file 1). Even without attempting to interpret the direction of event movements (shifts in events), certain developmental tendencies are recognizable. Because of our focus on the origin of Cladocera, we only compared the Onychocaudata, Cladoceromorpha and Cladocera nodes. Between Onychocaudata and Cladoceromorpha, the number of morphological events within the embryonic phase increases, while the number of morphological events in the larval phase decreases. The relationship between the two phases remains the same in Cladocera, but because a mandibular palpus and a nauplius eye is absent in *Penilia avirostris* the total number of morphological events is lower. Another significant trend is the reduction in the number of larval stages. The larval morphological events in Onychocaudata are spread over five stages (ACCTRAN; four stages in DELTRAN), while in Cladoceromorpha they take place in only four (ACCTRAN; three stages in DELTRAN) and in Cladocera (here Ctenopoda) only two larval stages are present (developmental phases color coded in Figure 7). In conclusion, it can be said that the reduced number of larval molts (and therefore the reduced number of pre-metamorphosis stages) in the developmental sequences of Cladoceromorpha and Cladocera lead to a greater ‘compression’ of morphological events within these sequences, i.e. to more events in a lower number of stages.

**Table 1:** List of embryonic and larval molts and stage values in the ancestral sequences

		**Embryonic events**	**Larval or embryo**-**like larval events**	**Pre**-**metamorphosis molts**	**Pre**-**metamorphosis stages**
Onychocaudata	ACC	4	17	4	5
	DEL	5	16	4	4
Cladoceromorpha	ACC	6	15	3	4
	DEL	6	15	3	3
Cladocera	ACC	6	13	1	2
	DEL	6	13	1	2

Although the Parsimov event-pairing analysis (Table 2, Additional file 2) revealed only a few unambiguous movements of morphological events, these shifts naturally tie in with our comparison of ancestral developmental sequences and make it easier to understand what we have concluded so far. Event-movements are observable in both in the ACCTRAN and DELTRAN optimizations and their consensus is mostly congruent. As in the comparison of developmental sequences, we only considered event-movements between the Onychocaudata node and the Cladoceromorpha node and between the latter and the node of crown-group Cladocera.

**Table 2:** **Morphogenetic event movements** (**only shifts of the consensus are listed**)

**Branch leading from the Onychocaudata to the common ancestor of the Cladoceromorpha**			
	**Morphogenetic event**	**Shift**	**Relative to…**
C/D	Naupliar SL-ir somata are expressed / moveable naupliar appendages are present	late	Hatching from resting or subitaneous egg (**A**), presence of a naupliar nervous system (**B**), telsonic longitudinal neurite bundles are present (**G**), first molt (**I**)
T	Differentiation of the ventral food groove	early	Postnaupliar SL-ir somata are expressed (**P**), moveable anterior trunk limbs are developed (**R**)
V	Third molt	late	Postnaupliar SL-ir somata are expressed (**P**), moveable anterior trunk limbs are developed (**R**)
**Branch leading from the Cladoceromorpha to the common ancestor of the Cladocera**			
	**Morphogenetic event**	**Shift**	**Relative to…**
C/D	Naupliar SL-ir somata are expressed / moveable naupliar appendages are present	late	Conical furcal rami are developed at the posterior end of the body (**N**), postnaupliar SL-ir somata are expressed (**P**), moveable anterior trunk limbs are developed (**R**), differentiated furcal rami are present at the posterior end of the body (**U**)
H	Carapace anlagen appear dorsally	late	Conical furcal rami are developed at the posterior end of the body (N), compound eye anlagen appear dorsally (**S**)
O	Second molt	late	Postnaupliar SL-ir somata are expressed (**P**), moveable anterior trunk limbs are developed (**R**), Differentiated furcal rami are present at the posterior end of the body (**U**)
T	Differentiation of the ventral food groove	early	postnaupliar neuromeres are developed (**K**), thoracic neuromeres are developed (**L**), three lateral appendage nerves are present (**Q**)
X	Anterior-most trunk limbs are almost completely differentiated	early	Postnaupliar SL-ir somata are expressed (**P**), three lateral appendage nerves are present (**Q**), moveable anterior trunk limbs are developed (**R**), differentiated furcal rami are present at the posterior end of the body (**U**), third molt (**V**)
Z	Optical neuropil anlagen appear in the protocerebrum	early	Differentiated furcal rami are present at the posterior end of the body (**U**), third molt (**V**)

A first step in the direction of the reduction in the number of pre-metamorphosis stages observed between the Onychocaudata node and the Cladoceromorpha node is the shift in the third molt (V) to a later position within the larval phase. A shift-related increase in the number of morphological events in the embryonic phase from the Onychocaudata to the Cladoceromorpha nodes could only be revealed in the DELTRAN optimized analysis, where the events visible thoracic limb rows (F), presence of buds of maxillulae and maxillae (J) and presence of primordial anterior trunk limbs (M) shifted to an earlier position relative to the hatching process (A) into the embryonic phase.

In addition, the overall analysis also shows a shift in functionality out of the embryonic phase and into the larval phase, as indicated by the later appearance of naupliar SL-ir expression (C) and moveable naupliar appendages (D).

The compression of morphological events in the larval phase in the transition from the Cladoceromorpha node to the Cladocera node is explained by a shift in events T, X and Z to an earlier position within this phase as a result of which the third molt (V) no longer serves to separate larval stages, at least with regard to the character set examined herein. The reduction in pre-metamorphosis stages is the result of a shift in the position of the second molt (O) to later on in the larval phase (ultimately to become a juvenile molt in Cladocera). The delay in functionality is caused by the events naupliar SL-ir expression (C) and the presence of moveable naupliar appendages (D) moving to an even later position relative to a suite of other events (arrows indicate event shifts in Figure 7). In general, all the event movements associated with the transitions to the nodes of Cladoceromorpha and Cladocera indicate earlier differentiation of morphological structures and delayed functionality (evidenced by the occurrence of SLI or appendage movements, for example). This corresponds well with the cladoceromorphan and cladoceran developmental strategy in which embryo-like larva develop under the protection of the dorsal carapace of the mother animal without having to perform feeding or swimming activities. Anamorphic indirect developers, on the other hand, are found swimming directly after hatching.

## Conclusion

All the event movements we were able to detect reveal heterochronous shifts towards the nodes of Cladoceromorpha and Cladocera that tie in on the whole with the findings of our previous study [60]. While the earlier study was based on a comparison of extant developmental sequences only, the approach taken herein enabled us to reveal (within the theoretical framework of parsimony) the direction of morphogenetic changes in the chronology of the ancestral developmental sequences of Branchiopoda, Phyllopoda, Diplostraca, Onychocaudata, Cladoceromorpha and Cladocera. The compression of morphological events within the embryonic and larval phases observed towards both the Cladoceromorpha and Cladocera nodes is caused by (1) a shift in larval events into the embryonic phase and (2) a shift in previously larval molts into the juvenile phase.

This compression of morphological events and reduction in the number of pre-metamorphosis molts might also be accompanied by a shorter and more rapid process of overall development than that of anamorphic indirect developers. Studies into a number of cladoceran species report rapid development until the first fertile juveniles appear (*Daphnia* juveniles, for example, are able to develop within 70 hours: [3]). No additional segments appear during the juvenile phase of Cladocera, and sexual maturity is soon reached [4]. In contrast, anamorphic indirect developers do not even possess a genital segment in the early juvenile stages. Furthermore, secondary sexual characters such as a specialized head or thoracic appendages (anostracan male pincer- shaped antennae, notostracan female oostegopods of the eleventh thoracopods, modified spinicaudatan female epipodites of the tenth and eleventh thoracopods to carry eggs) have to develop before reproduction can take place [4].

With regard to the theory of the progenetic evolutionary transformation of Cladocera from a conchostracan-like ancestor, our study failed to reveal precocious sexual maturation in an ancestral cladoceromorphan larva. Instead, our results indicate that the process was one of more gradual heterochronous evolution. Starting from an onychocaudatan ancestor, development switched firstly from anamorphic indirect to pseudo-direct (and direct) development in the cladoceromorphan ancestor. Simultaneously, previously larval events shifted into the embryonic phase, the ability to feed and swim appeared later in development and certain larval molts shifted into the juvenile phase, leading to an initial compression of morphological events in the larval phase. The morphology of Cladocera and simultaneously a further shift in larval molts into the juvenile stage, leading to an even greater compression of morphological events in the cladoceran larval phase, must have evolved directly in the lineage leading to the cladocerans, after the anamorphic developmental strategy was lost.

Nevertheless, the shift in morphological events into the embryonic phase, the reduction in embryo-like larval stages and the shortening of the juvenile phase ultimately did result in precocious maturation, such that some kind of progenesis did indeed occur, although not that suggested by Claus´ theory of cladoceran origin, which has a sexually mature free-swimming larva as its starting point [59].

Shortened development is the key to building up a new population rapidly and successfully. In the evolution of Cladoceromorpha, one might speculate, the shortening of the development period may also be responsible for the rapid and successful colonization of new habitats and can thus be regarded as preadaptation [14]. From an evolutionary standpoint, a reduction in the time needed for development gave both taxa the potentially beneficial means of colonizing newly established habitats more successfully and more rapidly and of adapting more easily to changing environmental conditions. Our conclusions converge with Gould´s (1977: 338) argument that 'progenesis is selected not primarily for morphology but by the need for precocious maturation as a life-history strategy'.

## Materials and methods

Collection, fixation and labeling procedures.

The *Penilia avirostris* material was obtained from the Gulmarsfjord at the 'Klubban' marine station of the University of Uppsala, Sweden. Planktonic samples were collected with a fine plankton net attached to a rope which was pulled through the marine sea layers of the fjord.

The samples were fixed for 30 min in a 4% Paraformaldehyde (PFA, 16% stem solution, Electron Microscopy Sciences) solution in 0.1 M PBS (pH 7.4) at room temperature.

Immunohistochemical labeling and data analysis were performed as described in Fritsch and Richter [95]. The nuclei were counterstained using Sytox Green (Molecular Probes, S-7020, dilution 1:600 in 0.1 M PBS). To label acetylated alpha tubulin, a primary monoclonal mouse antibody (clone 6–11 B-1, Sigma T6793) was used in combination with a Cy-3 coupled secondary goat antibody (affini pure anti-mouse IgG (H+L), Jackson Immunoresearch 155-165-003). To detect the serotonin expression pattern, a primary polyclonal rabbit antiserum (anti-serotonin, Sigma S5545) was used together with an Alexa-488 coupled secondary goat antibody (anti-rabbit IgG (H+L), Molecular Probes A-11008). The immunohistochemically labeled specimens were scanned with a Leica DMI6000 CFS confocal laser scanning microscope equipped with a conventional scanning system Leica TCS SP5 II, successive z-planes ranging from 0.4 to 1.0 μm. Unstained larval and juvenile material mounted on microscope slides and embedded in Vectashield® Mounting Medium (Vector Laboratories) was used to analyze the external morphology of the larval cuticle. To obtain a fluorescence signal from the larval cuticle that could be viewed with the confocal microscope, a 458 and 488 nm wavelength laser light was used. To analyze external and internal morphology, we used the 3D reconstruction software Imaris 7.0 (®Bitplane, Switzerland). The figure plates were created using the graphics software Coral Graphic Suite XIII (®Corel Corporation). The terminology of nervous system structures is in accordance with the glossary of Richter et al. [98].

### Comparing development

**Figure 8:**
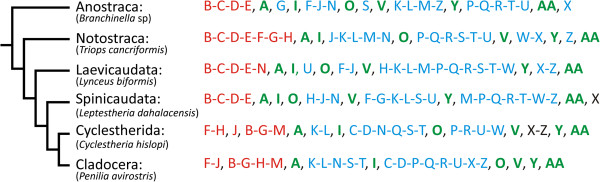
**Developmental sequences for the six branchiopod taxa analyzed: phylogeny of the Branchiopoda based on [**[6]**] and [**[30]**].** Red letters represent embryonic development and blue larval development. Green letters indicate the hatching and molting processes.

Comparing developmental data across different taxa is a process fraught with problems, one of the main ones being the arbitrary nature of classifications such as those based on age, size or stage, instar or stase [14,99-102]. These terms are used to arbitrarily describe a real, developmentally fixed condition, but only at the specific time of observation. Development does not cease or pause at a specific time though, it is a continuous process. Another shortcoming of comparisons using these developmental distinctions is their failure to take account of possible shifts in the chronology of the development, which effectively eradicate any absolute landmarks against which to make the comparisons. A huge amount of literature has revealed that in many cases, in vertebrate embryology for example e.g. [103-111], the development of structures may start at different relative times in different taxa (heterochrony [112,113]). To reveal any temporal shifts in development which may have come about during evolution, and to study similarities and differences in the ontogeny of the different branchiopod lines, we determined 27 morphogenetic events in the development of the external morphology and nervous system of representatives of Anostraca, Notostraca, Laevicaudata, Spinicaudata, Cyclestherida and Cladocera (Table 3). Like morphological structures, morphogenetic events are mostly subjectively defined. In our analysis the events relate to embryonic, larval, embryo-like larval and juvenile structures. The hatching process and the post-hatching molts are also included in the list. Because each larval stage interacts directly with the environment at least by its external characters, the molting is considered as a special kind of morphological event. A chronological developmental sequence of these pre-determined morphogenetic events was then drawn up for each of the six taxa, with the result providing a picture of the progression of ontogeny e.g. [104,105,107,114-116]. Within the chronological sequences each single morphogenetic event is represented by a letter. Events (letters) which appear consecutively are divided by a comma, regardless of the actual time interval between them, and events which appear simultaneously are separated by a dash. Chronological sequences consisting of 27 morphogenetic events for Anostraca, Notostraca, Laevicaudata, Spinicaudata, Cyclestherida and Cladocera are presented in Figure 8.

**Table 3:** **Tabular list of 27 embryonic**, **larval and juvenile morphogenetic events**

**Label**	**Event**
A	Hatching from resting or subitaneous egg
B	Presence of a naupliar nervous system
C	Naupliar SL-ir somata are expressed
D	Moveable naupliar appendages are present
E	Presence of a mandibular palp
F	Thoracic limb rows are visable
G	Telsonic longitudinal neurite bundles are present
H	Carapace analgen appear dorsally
I	First molt
J	Maxillulae and maxillae buds are present
K	Postnaupliar neuromeres are developed
L	Thoracic neuromeres are developed
M	Primordial anterior trunk limbs are present
N	Conical furcal rami are developed at the posterior end of the body
O	Second molt
P	Postnaupliar SL-ir somata are expressed
Q	Three lateral appendage nerves are present
R	Moveable anterior trunk limbs are developed
S	Compound eye anlagen appear dorsally
T	Differenatiation of the ventral food groove
U	Differentiated furcal rami are present at the posterior end of the body
V	Third molt
W	Four nauplius eye cups are present
X	Anterior-most trunk limbs are almost completely differentatiated
Y	Fourth molt
Z	Optical neuropil anlagen appear in the protocerebrum
AA	Fifth molt

Events relate to external morphological and internal nervous system structures. Hatching and molting processes are also included.

Simultaneous events in a developmental sequence are inherently problematic. While development is an ongoing process, many cell processes and organ formations take place sequentially and may even be dependent on each other. Processes (events) which occur at exactly the same time during ontogeny are unusual [105,106,108]. The developmental sequences we obtained from our investigations into the six branchiopod taxa are of a rather low chronological resolution before the hatching process and between the molts, with several events seemingly appearing at the same time. This is likely a methodological problem and not a reflection of the real situation, for although we investigated several individuals of each species at each stage, we still only obtained a specific, not necessarily representative picture of the ontogeny. A high-resolution chronology of development requires specimens to be investigated at very short periodic intervals (or the same specimen continuously through time), but even then there is no guarantee of complete resolution or of avoiding simultaneous artifacts. In addition, this higher level of resolution might also reveal effective simultaneity caused by natural intra-specific variation in the relative timing of two events that otherwise occur close together in time, where one event precedes the other in one individual, but vice versa in another.

### Methodology of parsimov event-pairing analysis

**Figure 9:**
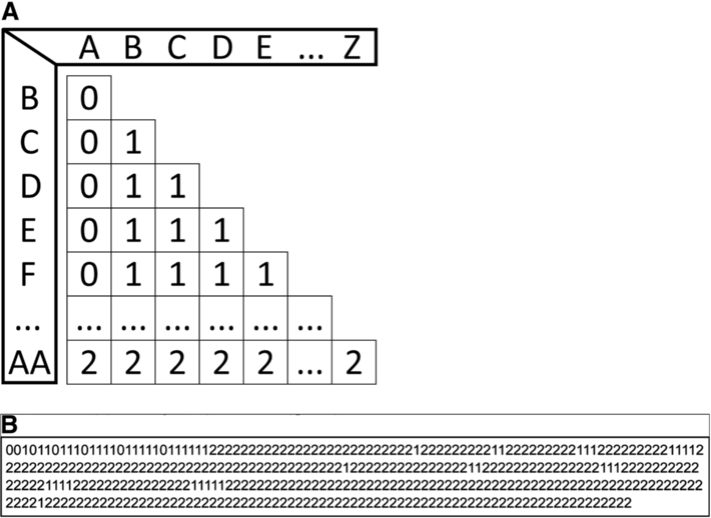
**Event-pairing methodology. A.** Event-pairing matrix in *Triops cancriformis*. **B.** Event-pairing code in *Triops cancriformis*

To compare and analyze the developmental sequences obtained we used an event-pairing approach. The relative timing of each of the 27 morphogenetic events (in each species) was compared to that of every other event to form a sequence unit *sensu* Velhagen [107]. This resulted in a matrix of 351 unique event-pairings (i.e., the relative timing of event B versus A is included but not A versus B); part of the *Triops cancriformis* matrix is represented in Figure 9A). The pairs can be regarded as character states which express the relative timing of one event to another. An event can occur before, simultaneously to or after another event e.g. [107-111,115]. According to these three possible temporal relations, event-pairings are scored 0, 1 or 2, respectively. Event pairs where information for one or both of the underlying events was missing are scored as question marks. By linearizing the matrix of all 351 event-pair scores we were able to obtain a sequential event-pairing code (that for *Triops cancriformis* is shown in Figure 9B). The same procedure was applied to each of the six species investigated.

To enable us to analyze and compare these datasets, the event-pairing codes thus obtained were entered into standard phylogenetic software such as WinClada [117] or PAUP [118]. Because the inherent non-independence of the event-pairing data (e.g., if event A precedes event B and B precedes C, then A must also precede C; despite this redundancy of information all three event pairs are coded nonetheless) might bias a phylogenetic analysis [108-111], we mapped the developmental event-pairing codes onto an existing phylogenetic framework (the branchiopod phylogeny used is based on [6] and [30]). By reconstructing the apomorphic changes at each node, we were thus able to identify potential temporal shifts in the chronology of development (heterochrony), something that can only be achieved by comparing developmental data in a phylogenetic framework [103].

This kind of analysis reveals specific transformations in the character states of an event-pair, which in turn reflect a rearrangement of the underlying events in the developmental sequence. However, it is not possible on the basis of this information alone to determine which of the underlying events moved to cause a change in an event-pair score, and the possibility of a shift in both cannot be ruled out either (i.e. no information is available on the direction of event-movements [110,111]). However, the non-moving events that can be identified by examining all event-pair transformations relative to one another effectively form the background by which to judge those events that have actually moved. Specifically, in order to reveal the polarity of event-movements, we adopted the **Parsimov event**-**pairing analysis** invented by Jeffery et al. (2005) to find 'the minimal solution that accounts for every event-pair change and it yield a consensus that contains all hypotheses of movement that must necessarily form part of any equally most parsimonious solution to the observed event-pair changes' (p. 239 in [111]). Using our initial developmental sequence data from extant representatives and this analytical method, we were able to reconstruct not only the consensus ancestral developmental sequences for each taxon, but also the most parsimonious event-movements over evolutionary time. To obtain the latter, we primarily used, conservatively, the suite of events that were inferred to have moved under both ACCTRAN and DELTRAN optimization of the event-pair data taken from the reference topology (Additional files 3, 4, 5, and 6).

## Competing interests

The authors declare that they have no competing interests.

## Authors’ contribution

MF and SR sampled the material and MF carried out the fixation, pretreatment and immunohistochemical procedures, took the confocal laser-scanning microscopic recordings, performed the data reconstruction analysis and designed the figures. The study was designed by MF and SR. The computational analysis of the Parsimov event-pairing program was conducted by OBE. The evaluation of the Parsimov event-pairing data and the main document was drafted by MF, the final version of the article was discussed and phrased with SR and OBE. All authors approved the final version of the manuscript.

## Supplementary Material

Additional file 1 Branchiopoda value list. Click here for file

Additional file 2 Branchiopoda morphogenetic movements. Click here for file

Additional file 3 Parsimov event-pairing analysis - ACCTRAN Ancestral Sequences. Click here for file

Additional file 4 Parsimov event-pairing analysis - ACCTRAN Parsimov shifts. Click here for file

Additional file 5 Parsimov event-pairing analysis - DELTRAN Ancestral Sequences. Click here for file

Additional file 6 Parsimov event-pairing analysis - DELTRAN Parsimov shifts. Click here for file

## References

[B1] Martin JW Harisson FW Branchiopoda Microscopic Anatomy of Invertebrates, Crustacea Vol 9 1992 New York: Wiley-Liss, Inc. 25 224

[B2] Smirnov NN Cladocera (Crustacea) from the Permian of eastern Kazakhstan Paleontol Z 1970 3 95 100

[B3] Flößner F Die Haplopoda und Cladocera Mitteleuropas 2000 Leiden: Backhuys

[B4] Dumont HJF Negrea SV Introduction to the class Branchiopoda 2002 Leiden: Backhuys

[B5] Korovchinsky NM The Cladocera (Crustacea: Branchiopoda) as a relict group. Zool J Linn Soc-Lond 2006 147 109 24 10.1111/j.1096-3642.2006.00217.x

[B6] Richter S Olesen J Wheeler WC Phylogeny of Branchiopoda (Crustacea) based on a combined analysis of morphological data and six molecular loci Cladistics 2007 23 301 36 10.1111/j.1096-0031.2007.00148.x 34905837

[B7] Eriksson S Studien über die Fangapparate der Branchiopoden nebst einiger phylogenetischer Bemerkungen Zoologiska Bidr Upps 1934 15 24 287

[B8] Huxley J Hardy AC Ford EB Escape from specialization Evolution as a progress 1954 London: George Allen & Unwin Ltd 122 42

[B9] Remane A Die Grundlagen des Natürlichen Systems der Vergleichenden Anatomie und der Phylogenetik 1956 Leipzig: Geest & Portig

[B10] Remane A Zur Frage der Neotenie und Fetalisation Zool Anz 1960 164 417 20

[B11] Schminke HK Adaptation of Bathynellacea (Crustacea, Syncarida) to life in the interstitial ("Zoea theory") Int Rev Ges Hydrobiol Hydrograph 1981 66 575 637 10.1002/iroh.19810660411

[B12] Fryer G A new classification of the branchiopod Crustacea Zool J Linn Soc-Lond 1987 91 357 83 10.1111/j.1096-3642.1987.tb01420.x

[B13] Giard A La Castration parasitaire et son influence sur les caractères extérieurs du sexe mâle chez les Crustacés décapodes, par le professeur Bull Biol France Belg 1887 18 1 28

[B14] Gould SJ Ontogeny and Phylogeny 1977 Cambridge, Mass: Belknap Press

[B15] McKinney ML McNamara KJ Heterochrony - The evolution of ontogeny 1991 New York: Plenum Press

[B16] Kollmann J Das Ueberwintern von europäischen Frosch- und Tritonlarven und die Umwandlung des mexikanischen Axolotl Verh Naturf Ges Basel 1885 7 387 98

[B17] Garstang W The theory of recapitulation: a critical restatement of the biogenetic law J Linn Soc Zool 1922 35 81 101 10.1111/j.1096-3642.1922.tb00464.x

[B18] Garstang W The morphology of the Tunicata, and its bearing on the phylogeny of the Chordata Q J Microsc Sci 1928 72 51 187

[B19] Garstang W The morphology and relations of the Siphonophora Q J Microsc Sci 1946 87 103 93

[B20] Botnariuc N Viña Bayés N Orghidan T Contribution à La connaissance de la biologie de *Cyclestheria hislopi* (Baird), (Conchostraca: Crustacea)de Cuba Résultats des expéditions biospéléogiques Cubano-Roumaines á Cuba 1977 Bucuresti: Editura Academiei Republicii Socialiste Romania 257 262

[B21] Negrea S Botnariuc N Dumont HJ Phylogeny, evolution and classification of the Branchiopoda (Crustacea) Hydrobiologia 1999 412 191 212

[B22] Löpmann A Die Zweiäugikeit von *Diaphanosoma* (Zugleich ein Beitrag zur Kenntnis des Cladocerenauges) Int Rev Ges Hydrobio 1937 34 432 84 10.1002/iroh.19370340123

[B23] Löpmann A Beitrag zum Problem der Phylogenie der Cladoceren Int Rev Ges Hydrobiol Hydrograph 1940 40 361 67 10.1002/iroh.19400400502

[B24] Fryer G A comment on a recent phylogenetic analysis of certain orders of the branchiopod Crustacea Crustaceana 1999 72 1039 1050 10.1163/156854099504004

[B25] Fryer G The elucidation of branchiopod phylogeny Crustaceana 2001 74 105 14 10.1163/156854001505389

[B26] Fryer G Branchiopod phylogeny: Facing the facts Crustaceana 2002 75 85 88 10.1163/156854002317373546

[B27] Martin JW Cash-Clark CE The external morphology of the onychopod 'cladoceran' genus Bythotrephes (Crustacea, Branchiopoda, Onychopoda, Cercopagididae), with notes on the morphology and phylogeny of the order Onychopoda Zool Scr 1995 24 61 90 10.1111/j.1463-6409.1995.tb00475.x

[B28] Olesen J A phylogenetic analysis of the Conchostraca and Cladocera (Crustacea, Branchiopoda, Diplostraca) Zool J Linn Soc-Lond 1998 122 491 536 10.1111/j.1096-3642.1998.tb02161.x

[B29] Olesen J Monophyly and phylogeny of Branchiopoda, with focus on morphology and homologies of branchiopod phyllopodous limbs J Crustacean Biol 2007 27 165 83 10.1651/S-2727.1

[B30] Olesen J Phylogeny of Branchiopoda (Crustacea) - character evolution and contribution of uniquely preserved fossils Arthropod Systematics & Phylogeny 2009 67 3 3923802466

[B31] de Waard JR Sacharova V Cristescu MEA Remigio EA Crease TJ Hebert PDN Probing the relationships of the branchiopod crustaceans Mol Phylogenet Evol 2006 39 491 502 10.1016/j.ympev.2005.11.003 16406819

[B32] Stenderup JT Olesen J Glenner H Molecular phylogeny of the Branchiopoda (Crustacea) - multiple approaches suggest a 'diplostracan' ancestry of the Notostraca Mol Phylogenet Evol 2006 41 182 94 10.1016/j.ympev.2006.06.006 16904916

[B33] Schwentner M Clavier S Fritsch M Padhye S Timms B Richter S *Cyclestheria hislopi* (Crustacea: Branchiopoda): a group of morphologically cryptic species with origins in the Cretaceous Mol Phylogenet 66 800 810 submitted 10.1016/j.ympev.2012.11.005 23178560

[B34] Ax P Das System Der Metazoa II 1999 Stuttgart, Jena, Lübeck, Ulm: Gustav Fischer Verlag

[B35] Braband A Richter S Hiesel R Scholtz G Phylogenetic relationships within the Phyllopoda (Crustacea, Branchiopoda) based on mitochondrial and nuclear markers Mol Phylogenet Evol 2002 25 229 44 10.1016/S1055-7903(02)00253-1 12414306

[B36] Olesen J Richter S Onychocaudata (Branchiopoda: Diplostraca), a new high-level taxon in branchiopod systematics J Crustacean Biol 2013 33 62 65 10.1163/1937240X-00002121

[B37] Regier JC Shultz JW Zwick A Hussey A Ball B Wetzer R Martin JW Cunningham CW Arthropod relationships revealed by phylogenomic analysis of nuclear protein-coding sequences Nature 2010 463 1079 83 10.1038/nature08742 20147900

[B38] von Reumont BM Jenner RA Wills MA Dell´Ampio E Pass G Ebersberger I Meyer B Koenemann S Iliffe TM Stamatakis A Niehuis O Meusemann K Pancrustacean Phylogeny in the light of new phylogenomic data Mol Biol 2010 l29 1031 45 10.1093/molbev/msr270 22049065

[B39] Flößner F Dahl F Kiemen- Und Blattfüßer, Branchiopoda; Fischläuse, Branchiura 1972 Die Tierwelt Deutschlands Jena: Fischer

[B40] Rivier IK The predatory Cladocera (Onychopoda: Podonidae, Polyphemidae, Cercopagidae) and Leptodorida of the world 1998 Leiden: Backhuys

[B41] Fryer G Diapause, a potent force in the evolution of freshwater crustaceans Hydrobiologia 1996 320 1 14 10.1007/BF00016800

[B42] Alekseev VR De Stasio B Gilbert JJ Diapause in aquatic invertebrates: Theory and human use 2007 Dordrecht: Springer

[B43] Benesch R Zur Ontogenie und Morphologie von *Artemia salina* L Zool Jahrb Abt Anat B 1969 86 307 458

[B44] Kotov AA Boikova OS Study of the late embryogenesis of *Daphnia* (Anomopoda, 'Cladocera', Branchiopoda) and a comparison of development in Anomopoda and Ctenopoda Hydrobiologia 2001 442 127 43 10.1023/A:1017594402114

[B45] Sassaman C Sex-Ratio variation in female-biased populations of notostracans Hydrobiologia 1991 212 169 79 10.1007/BF00025998

[B46] Zaffagnini F Rudimentary hermaphroditism and automictic parthenogenesis in *Limnadia lenticularis* (Phyllopoda Conchostraca) Experientia 1969 25 650 651 10.1007/BF01896572 5800140

[B47] Sassaman C Sex determination and evolution of unisexuality in the Conchostraca Hydrobiologia 1995 298 45 65 10.1007/BF00033799

[B48] Weeks SC Chapman EG Rogers DC Senyo DM Hoeh WR Evolutionary transitions among dioecy, androdioecy and hermaphroditism in limnadiid clam shrimp (Branchiopoda: Spinicaudata) J Evolution Biol 2009 22 1781 99 10.1111/j.1420-9101.2009.01813.x 19702888

[B49] Boikova OS Comparative investigation of the later embryogenesis of *Leptodora kindtii* (Focke, 1844) (Crustacea: Branchiopoda), with notes on types of embryonic development and larvae in Cladocera J Nat His 2008 42 2389 2416 10.1080/00222930802277590

[B50] Olesen J Scholtz G On the ontogeny of the Branchiopoda (Crustacea): Contribution of development to phylogeny and classification Crustacean Issues Vol 15 2004 AA Balkema: Lisse, Abingdon, Exton (PA), Tokyo 217 69

[B51] Ingle R Larval Stages of Northeastern Atlantic Crabs 1991 First Edition ed: Springer

[B52] Brusca RC Brusca GJ Invertebrates. Second 2002 Edition Sunderland: Sinauer Associates

[B53] Whitington PM Scholtz G The development of the crustacean nervous system Crustacean Issues Vol 15 2004 AA: Balkema 135 67

[B54] Scourfield DJ On a new type of crustacean from the old red sandstone (Rhynie chert bed, Aberdeenshire) - *Lepidocaris rhyniensis*, gen. et sp. nov Philos T Roy Soc B 1926 214 153 87 10.1098/rstb.1926.0005

[B55] Scourfield DJ Two new and nearly complete specimens of young stages of the Devonian fossil crustacean *Lepidocaris rhyniensis* J Zool Linn Soc-Lond 1940 152 290 98

[B56] Walossek D The Upper Cambrian *Rehbachiella* and the phylogeny of Branchiopoda and Crustacea Fossils and Strata 1993 32 1 202

[B57] Waloßek D The upper Cambrian *Rehbachiella*, its larval development, morphology and significance for the phylogeny of Branchiopoda and Crustacea Hydrobiologia 1995 298 1 13 10.1007/BF00033797

[B58] Haug C Haug JT Olesen J Martin JW, Olesen J, Høeg J Uniquely preserved fossil larvae, some with branchiopod affinities, from the Devonian: the Rhynie and Windyfield cherts 2013 Atlas of Crustacean Larvae: Johns Hopkins University Press

[B59] Claus C Untersuchungen Zur Erforschung Der Genealogischen Grundlage des Crustaceen-Systems Verlag Carl Gerold´s Sohn 1876 Wien

[B60] Fritsch M Richter S Nervous system development in Spinicaudata and Cyclestherida (Crustacea, Branchiopoda) - comparing two different modes of indirect development by using an event pairing approach J Morphol 2012 273 672 695 10.1002/jmor.20014 22460765

[B61] Olesen J Larval and post-larval development of the branchiopod clam shrimp *Cyclestheria hislopi* (Baird, 1859) (Crustacea, Branchiopoda, Conchostraca, Spinicaudata) Acta Zool Stockholm 1999 80 163 84 10.1046/j.1463-6395.1999.80220015.x

[B62] Sars GO On Cyclestheria hislopi (Baird), a new generic type of bivalve Phyllopoda raised from the dried Australian mud Videnskabs-Selskabs Forhandlinge 1887

[B63] Kotov AA Fate of the second maxilla during embryogenesis in some Anomopoda Crustacea (Branchiopoda) Zool J Linn Soc-Lond 1996 116 393 405 10.1111/j.1096-3642.1996.tb00130.x

[B64] Kotov AA Boikova OS Comparative analysis of the late embryogenesis of *Sida crystallina* (O.F. Müller, 1876) and *Diaphanosoma brachyurum* (Lievin, 1848) (Crustacea: Branchiopoda: Ctenopoda) Hydrobiologia 1998 380 103 25 10.1023/A:1003269331645

[B65] Olesen J Richter S Scholtz G On the ontogeny of *Leptodora kindtii* (Crustacea, Branchiopoda, Cladocera), with notes on the phylogeny of the Cladocera J Morphol 2003 256 235 59 10.1002/jmor.10043 12655608

[B66] Olesen J Grygier MJ Larval development of Japanese "conchostracans": Part 1, larval development of *Eulimnadia braueriana* (Crustacea, Branchiopoda, Spinicaudata, Limnadiidae) compared to that of other limnadiids Acta Zool-Stockholm 2003 84 41 61

[B67] Roessler EW Review of Colombian Conchostraca (Crustacea) - ecological aspects and life cycles - family Cyclestheriidae Hydrobiologia 1995 298 113 24 10.1007/BF00033806

[B68] Egloff A Fofonoff PW Onbe T Reproductive biology of marine cladocerans Adv Mar Biol 1997 31 79 167

[B69] Sars GO Om en dimorph Udvikling samt Generationsvexel hos Leptodora Videnskabs- Selskabs Forhandlinger 1873

[B70] Warren HS The central nervous system of the adult *Artemia*." T Am Microsc Soc 1930 49 189 209 10.2307/3222346

[B71] Gerberding M Germ band formation and early neurogenesis of *Leptodora kindtii* (Cladocera): First evidence for neuroblasts in the entomostracan crustaceans Invertebr Reprod Dev 1997 32 63 73 10.1080/07924259.1997.9672605

[B72] Olesen J Richter S Scholtz G On the ontogeny of *Leptodora kindtii* (Crustacea, Branchiopoda, Cladocera), with notes on the phylogeny of the Cladocera J Morpho 2003 256 235 259 10.1002/jmor.10043 12655608

[B73] Korovchinsky NM Boikova OS Study of the external morphology of *Leptodora kindtii* Focke, 1844 (Crustacea: Branchiopoda: Haplopoda), with notes on its relation to Cladocera and on conspecificity of populations of the species over the Eurasian range J Nat Hist 2008 42 2825 2863 10.1080/00222930801919259

[B74] Gibitz A Verbreitung und Abstammung mariner Cladoceren Verh Zool-Bot Ges Wien 1922 71 85 105

[B75] Lochhead JH On the distribution of a marine cladoceran, *Penilia avirostris* Dana (Crustacea, Branchiopoda), with a note on its reported bioluminescence Biol Bull 1954 107 92 105 10.2307/1538633

[B76] Olesen J Richter S Scholtz G The evolutionary transformation of phyllopodous to stenopodous limbs in the Branchiopoda (Crustacea) - is there a common mechanism for early limb development in arthropods? Int J Dev Biol 2001 45 869 76 11804030

[B77] Michels J Confocal laser scanning microscopy: using cuticular autofluorescence for high resolution morphological imaging in small crustaceans J Microsc 2007 227 1 7 10.1111/j.1365-2818.2007.01787.x 17635653

[B78] Fritsch M Kaji T Olesen J Richter S The development of the nervous system in Laevicaudata (Crustacea, Branchiopoda) – insights into the evolution and homologies of branchiopod limbs and “frontal organs” Zoomorphology 2013 132 163 181 10.1007/s00435-012-0173-0

[B79] Grobben K Die Entwicklungsgeschichte der *Moina rectirostris* - Zugleich ein Betrag zur Kenntnis der Anatomie der Phyllopoden." Arb Zool Inst Univ Wien 1879 2 203 68

[B80] Kotov AA A special moult after the release of the embryo from the brood pouch of Anomopoda (Branchiopoda, Crustacea): A return to an old question Hydrobiologia 1997 354 83 87 10.1023/A:1003063407127

[B81] Sudler MT The development of *Penilia schmackeri* Richard Proc Soc Nat His Boston 1899 6 107 31

[B82] Valentin JL Maraozzo A Embryonic development of *Penilia avirostris* Dana, 1852 in a tropical bay in Brazil Braz J Biol 2004 64 891 894 15744431 10.1590/s1519-69842004000500020

[B83] Boikova OS Postembryonic development in *Diaphanosoma brachyurum* (Lievin, 1848) (Crustacea: Ctenopoda: Sididae) Hydrobiologia 2005 537 7 14 10.1007/s10750-004-1570-2

[B84] Atienza D Seiz E Skovgaard A Trepat I Calbet A Life history and population dynamics of the marine cladoceran *Penilia avirostris* (Branchiopoda: Cladocera) in the Catalan Sea (NW Mediterranean) J Plankton Res 2008 30 345 57 10.1093/plankt/fbm109

[B85] Weissmann A Über Bau und Lebenserscheinungen von *Leptodora hyalina* Z wiss Zool 1874 24 1 70

[B86] Samter M Studien zur Entwicklungsgeschichte der *Leptodora hyalina* Lillj Z wiss Zool 1900 68 169 260

[B87] Kirsch R Richter S The nervous system of *Leptodora kindtii* (Branchiopoda, Cladocera) surveyed with confocal scanning microscopy (cLSM), including general remarks on the branchiopod neuromorphological ground pattern Arthropod Struct Dev 2007 36 143 56 10.1016/j.asd.2006.08.013 18089095

[B88] Petrov B Larval development of *Leptestheria saetosa* Marincek and Petrov, 1992 (Leptestheriidae, Conchostraca, Crustacea) Arch Biol Sci Belgrade 1992 44 229 41

[B89] Eder E SEM investigations of the larval development of *Imnadia yeyetta* and *Leptestheria dahalacensis* (Crustacea: Branchiopoda: Spinicaudata) Hydrobiologia 2002 486 39 47 10.1023/A:1021322028552

[B90] Møller OS Olesen J Høeg JT SEM studies on the early larval development of *Triops cancriformis* (Bosc) (Crustacea: Branchiopoda, Notostraca) Acta Zool Stockholm 2003 84 267 84 10.1046/j.1463-6395.2003.00146.x

[B91] Møller OS Olesen J Høeg JT On the larval development of *Eubranchipus grubii* (Crustacea, Branchiopoda, Anostraca), with notes on the basal phylogeny of the Branchiopoda Zoomorphology 2004 123 107 23 10.1007/s00435-003-0093-0

[B92] Aramant R Elofsson R Distribution of monoaminergic neurons in the nervous system of non-malacostracan crustaceans Cell Tiss Res 1976 166 1 24 10.1007/BF00215121 1248033

[B93] Harzsch S Waloszek D Serotonin-immunoreactive neurons in the ventral nerve cord of Crustacea: A character to study aspects of arthropod phylogeny Arthropod Sruct Dev 2000 29 307 22 10.1016/S1467-8039(01)00015-9 18088936

[B94] Harzsch S Glötzner J An immunohistochemical study of structure and development of the nervous system in the brine shrimp *Artemia salina* Linnaeus, 1758 (Branchiopoda, Anostraca) with remarks on the evolution of the arthropod brain Arthropod Struct Dev 2002 30 251 70 10.1016/S1467-8039(02)00012-9 18088960

[B95] Fritsch M Richter S The formation of the nervous system during larval development in *Triops cancriformis* (Bosc) (Crustacea, Branchiopoda): An immunohistochemical survey J Morphol 2010 271 1457 1481 10.1002/jmor.10892 20938985

[B96] Hoshi T Studies on physiology and ecology of plankton VI glycogen in embryonic life of *Simocephalus vetulus*, with some notes on the energy source of development Biol Repts Tohoku Univ 4th ser 1951 19 123 33

[B97] Kitching I Forey P Humphries C Williams D Cladistics - Theory and practice of parsimony analysis 1998 Oxford: University Press Inc 70 78

[B98] Richter S Loesel R Purschke G Schmidt-Rhaesa A Scholtz G Stach T Vogt L Wanninger A Brenneis G Döring C Faller S Fritsch M Grobe P Heuer CM Kaul S Møller OS Müller CHG Rothe BH Stegner MEJ Harzsch S Invertebrate neurophylogeny: suggested terms and definitions for a neuroanatomical glossary Front Zool 2010 29 1 49 10.1186/1742-9994-7-29 PMC2996375 21062451

[B99] Alberch P Gould SJ Oster GF Wake DB Size and shape in ontogeny and phylogeny Paleobiology 1979 5 296 317

[B100] André HM Humphries CJ Age-dependent evolution: From theory to practice Ontogeny and systematics 1988 New York: Columbia University Press 137 87

[B101] Anger K Anger K The Biology of the decapod crustacean larvae Crustacean Issues Vol 14 2001 Rotterdam: AA Balkema 13 40

[B102] von Lieven AM The embryonic moult in diplogastrids (Nematoda) - homology of developmental stages and heterochrony as a prerequisite for morphological diversity Zool Anz 2005 244 79 91 10.1016/j.jcz.2005.05.001

[B103] Mabee PM Trendler TA Development of the cranium and paired fins in *Betta splendens* (Teleostei: Percomorpha): Intraspecific variation and interspecific comparisons J Morphol 1996 227 249 87 10.1002/(SICI)1097-4687(199603)227:3<249::AID-JMOR1>3.0.CO;2-1 29852576

[B104] Smith KK Integration of craniofacial structures during development in mammals Am Zool 1996 36 70 79

[B105] Smith KK Comparative patterns of craniofacial development in eutherian and metatherian mammals Evolution 1997 51 1663 78 10.2307/2411218 28568626

[B106] Nunn CL Smith KK Statistical analyses of developmental sequences: The craniofacial region in marsupial and placental mammals Am Nat 1998 152 82 101 10.1086/286151 18811403

[B107] Velhagen WA Analyzing developmental sequences using sequence units Syst Biol 1997 46 204 10 10.1093/sysbio/46.1.204 11975352

[B108] Bininda-Emonds ORP Jeffery JE Coates MI Richardson MK From Haeckel to event- pairing: the evolution of developmental sequences Theor Biosci 2002 121 297 320

[B109] Jeffery JE Richardson MK Coates MI Bininda-Emonds ORP Analyzing evolutionary patterns in amniote embryonic development Evol Dev 2002 4 292 302 10.1046/j.1525-142X.2002.02018.x 12168621

[B110] Jeffery JE Bininda-Emonds ORP Coates MI Richardson MK Analyzing developmental sequences within a phylogenetic framework Syst Biol 2002 51 478 91 10.1080/10635150290069904 12079645

[B111] Jeffery JE Bininda-Emonds ORP Coates MI Richardson MK A new technique for identifying sequence heterochrony Syst Biol 2005 54 230 40 10.1080/10635150590923227 16012094

[B112] Richardson MK Jeffery JE Costes MI Bininda-Emonds ORP Comparative methods in developmental biology Zoology 2001 104 278 83 10.1078/0944-2006-00033 16351842

[B113] McKinney ML McNamara KJ Heterochrony - The evolution of ontogeny Plenum Press 1991

[B114] Alberch P Problems with the interpretation of developmental sequences Syst Zool 1985 34 46 58 10.2307/2413344

[B115] Smith KK Heterochrony revisited: the evolution of developmental sequences Biol J Linn Soc 2001 73 169 86 10.1111/j.1095-8312.2001.tb01355.x

[B116] Smith KK Sequence heterochrony and the evolution of development J Morphol 2002 252 82 97 10.1002/jmor.10014 11921037

[B117] Nixon KC Winclada (BETA) ver. 0.9.9 1999 NY: ITHACA

[B118] Swofford D PAUP*: phylogenetic analysis using parsimony (*and other methods), version 4 2002 Sunderland, MA: Sinauer

