# Supplementary material for: Unraveling the origin of Cladocera by identifying heterochrony in the developmental sequences of Branchiopoda
Source: Front Zool. 2013 Jun 19;10:35. doi: 10.1186/1742-9994-10-35 (PMC3716531; doi:10.1186/1742-9994-10-35)
Supplement: Additional file 4 — Parsimov event-pairing analysis - ACCTRAN Parsimov shifts. [file 1742-9994-10-35-S4.rtf]

Parsimov event-pairing analysis - ACCTRAN Parsimov shifts


Summary of ASCAS-cracked data from "apoacctran_morph.txt"
Created by Parsimov 1.0.7g beta

For each node, a record of all the shortest
runs has been saved as a .out file.
These files have all have the suffix "-%262"

All characters used.  Exhaustive searches used if possible.
AccTran character-state optimization used

Original Tree:

                   /---------------------------------------------------------------------------------------- Anostraca
                   |
 -----------------11                /----------------------------------------------------------------------- Notostraca
                   |                |
                   \---------------10                 /----------------------------------------------------- Laevicaudata
                                    |                 |
                                    \-----------------9                 /----------------------------------- Spinicaudata
                                                      |                 |
                                                      \-----------------8                /------------------ Cyclestherida
                                                                        \----------------7
                                                                                         \------------------ Cladocera

======================================
Node 11 --> Anostraca
6 characters with informative movement
Thorough search using all possible unpolarised seed-combinations (720 iterations)

Consensus of 1 MS solution without conflicts
Consensus has 3/3 steps (100.0%)

Twins (6, 14)
Char 7 moved L relative to 1, 2
Char 19 moved E relative to 20, 21

======================================
Node 11 --> Node 10
14 characters with informative movement
Heuristic search using three unpolarised seeds (2184 iterations)

Consensus of 16 MS solutions without conflicts
Consensus has 4/6 steps (66.7%)

Twins (6, 7)
Char 19 moved L relative to 11, 12, 13, 16, 17, 18
Char 25 moved L relative to 16, 17, 18, 20
Char 26 moved L relative to 11, 12, 13, 16, 17, 18, 20, 25

======================================
Node 10 --> Notostraca
25 characters with informative movement
Heuristic search using three unpolarised seeds (13800 iterations)

Consensus of 16 MS solutions without conflicts
Consensus has 9/9 steps (100.0%)

Char 6 moved E relative to 1, 2, 3, 4, 5, 9, 10, 14
Char 7 moved E relative to 3, 4, 5, 8
Char 8 moved E relative to 9, 10, 15
Char 11 moved E relative to 10, 14, 15, 19
Char 12 moved E relative to 10, 14, 15, 19
Char 13 moved E relative to 10, 14, 15
Char 22 moved L relative to 16, 17, 18, 20
Char 23 moved L relative to 16, 17, 18, 20
Char 24 moved E relative to 25, 26

======================================
Node 10 --> Node 9
17 characters with informative movement
Heuristic search using three unpolarised seeds (4080 iterations)

Consensus of 7 MS solutions without conflicts
Consensus has 7/8 steps (87.5%)

Twins (22, 19) (23, 24)
Char 8 moved L relative to 1, 2, 3, 4, 5, 6
Char 13 moved L relative to 16, 17, 18, 20
Char 14 moved E relative to 3, 4
Char 15 moved E relative to 6, 10
Char 21 moved E relative to 16, 17, 18, 20

======================================
Node 9 --> Laevicaudata
20 characters with informative movement
Heuristic search using three unpolarised seeds (6840 iterations)

Consensus of 101 MS solutions without conflicts
Consensus has 5/7 steps (71.4%)

Char 8 moved L relative to 11, 12, 13, 16, 17, 18, 20, 22
Char 11 moved L relative to 16, 17, 18, 20
Char 12 moved L relative to 16, 17, 18, 20
Char 14 moved E relative to 1, 2, 5, 9, 10
Char 21 moved E relative to 6, 10, 15

======================================
Node 9 --> Node 8
8 characters with informative movement
Heuristic search using three unpolarised seeds (336 iterations)

Consensus of 1 MS solution without conflicts
Consensus has 3/3 steps (100.0%)

Char 13 moved E relative to 11, 12
Char 19 moved E relative to 16, 17, 18
Char 26 moved E relative to 16, 17, 18

======================================
Node 8 --> Spinicaudata
26 characters with informative movement
Heuristic search using three unpolarised seeds (15600 iterations)

Consensus of 211 MS solutions without conflicts
Consensus has 9/12 steps (75.0%)

Char 6 moved L relative to 8, 10, 11, 12
Char 7 moved L relative to 1, 2, 9, 11, 12, 14, 15
Char 8 moved L relative to 14
Char 10 moved L relative to 8
Char 13 moved L relative to 11, 12
Char 14 moved L relative to 3, 4, 15
Char 21 moved E relative to 11, 12
Char 22 moved E relative to 21
Char 25 moved E relative to 16, 17, 18

======================================
Node 8 --> Node 7
25 characters with informative movement
Heuristic search using three unpolarised seeds (13800 iterations)

Consensus of 1986 MS solutions without conflicts
Consensus has 7/14 steps (50.0%)

Char 3 moved L relative to 1, 2, 7, 9
Char 4 moved L relative to 1, 2, 7, 9
Char 13 moved E relative to 16, 17, 18, 20
Char 20 moved E relative to 16, 18
Char 21 moved L relative to 16, 18, 23
Char 22 moved L relative to 16, 18, 23
Char 25 moved L relative to 24, 26

======================================
Node 7 --> Cyclestherida
15 characters with informative movement
Heuristic search using three unpolarised seeds (2730 iterations)

Consensus of 30 MS solutions without conflicts
Consensus has 5/7 steps (71.4%)

Char 14 moved L relative to 11, 12
Char 17 moved E relative to 16, 18, 21
Char 19 moved L relative to 3, 4, 11, 12
Char 20 moved L relative to 3, 4
Char 26 moved L relative to 16, 18

======================================
Node 7 --> Cladocera
20 characters with informative movement
Heuristic search using three unpolarised seeds (6840 iterations)

Consensus of 46 MS solutions without conflicts
Consensus has 8/8 steps (100.0%)

Char 3 moved L relative to 14, 16, 18, 21
Char 4 moved L relative to 14, 16, 18, 21
Char 8 moved L relative to 2, 7, 13
Char 9 moved L relative to 14, 19
Char 15 moved L relative to 16, 18, 21
Char 20 moved E relative to 11, 12, 17
Char 24 moved E relative to 16, 17, 18, 21, 22
Char 26 moved E relative to 21, 22

======================================

Run took 0d, 0h, 22m, 42s
