# Supplementary material for: Unraveling the origin of Cladocera by identifying heterochrony in the developmental sequences of Branchiopoda
Source: Front Zool. 2013 Jun 19;10:35. doi: 10.1186/1742-9994-10-35 (PMC3716531; doi:10.1186/1742-9994-10-35)
Supplement: Additional file 5 — Parsimov event-pairing analysis - DELTRAN Ancestral Sequences. [file 1742-9994-10-35-S5.rtf]

Parsimov event-pairing analysis - DELTRAN Ancestral Sequences

Node: 7 - Cladocera

Event: 1	Rank: 7	Min: 7	Max: 7	Conflict: 0
Event: 2	Rank: 4	Min: 3	Max: 6	Conflict: 3
Event: 3	Rank: 12	Min: 12	Max: 17	Conflict: 2
Event: 4	Rank: 12	Min: 12	Max: 17	Conflict: 2
Event: 5	Rank: 15	Min: 15	Max: 15	Conflict: 0
Event: 6	Rank: 2	Min: 1	Max: 3	Conflict: 2
Event: 7	Rank: 5	Min: 4	Max: 6	Conflict: 2
Event: 8	Rank: 3	Min: 2	Max: 4	Conflict: 2
Event: 9	Rank: 9	Min: 9	Max: 9	Conflict: 2
Event: 10	Rank: 1	Min: 1	Max: 2	Conflict: 1
Event: 11	Rank: 9	Min: 9	Max: 14	Conflict: 3
Event: 12	Rank: 9	Min: 9	Max: 14	Conflict: 3
Event: 13	Rank: 6	Min: 4	Max: 22	Conflict: 3
Event: 14	Rank: 12	Min: 8.5	Max: 16	Conflict: 6
Event: 15	Rank: 18	Min: 18	Max: 18	Conflict: 0
Event: 16	Rank: 20.5	Min: 17	Max: 22	Conflict: 3
Event: 17	Rank: 17	Min: 12	Max: 22	Conflict: 7
Event: 18	Rank: 20.5	Min: 17	Max: 22	Conflict: 3
Event: 19	Rank: 14	Min: 9	Max: 16	Conflict: 4
Event: 20	Rank: 16	Min: 12	Max: 22	Conflict: 4
Event: 21	Rank: 19	Min: 17	Max: 20.5	Conflict: 3
Event: 22	Rank: 23	Min: 21.5	Max: 23	Conflict: 1
Event: 23	Rank: 22	Min: 6	Max: 23.5	Conflict: 6
Event: 24	Rank: 24.5	Min: 24.5	Max: 24.5	Conflict: 0
Event: 25	Rank: 26	Min: 26	Max: 26	Conflict: 0
Event: 26	Rank: 24.5	Min: 24.5	Max: 24.5	Conflict: 0
Event: 27	Rank: 27	Min: 27	Max: 27	Conflict: 0

Node: 8 - Cladoceromorpha

Event: 1	Rank: 6	Min: 6	Max: 9	Conflict: 3
Event: 2	Rank: 4	Min: 2	Max: 8.5	Conflict: 5
Event: 3	Rank: 2	Min: 2	Max: 4	Conflict: 1
Event: 4	Rank: 2	Min: 2	Max: 4	Conflict: 1
Event: 5	Rank: 2	Min: 2	Max: 4	Conflict: 1
Event: 6	Rank: 10.5	Min: 6	Max: 10.5	Conflict: 4
Event: 7	Rank: 6	Min: 4	Max: 10.5	Conflict: 6
Event: 8	Rank: 8.5	Min: 4	Max: 12.5	Conflict: 6
Event: 9	Rank: 6	Min: 6	Max: 6	Conflict: 2
Event: 10	Rank: 8.5	Min: 5.5	Max: 10.5	Conflict: 4
Event: 11	Rank: 15.5	Min: 13.5	Max: 17	Conflict: 2
Event: 12	Rank: 15.5	Min: 13.5	Max: 17	Conflict: 2
Event: 13	Rank: 17	Min: 15.5	Max: 21	Conflict: 3
Event: 14	Rank: 10.5	Min: 8.5	Max: 10.5	Conflict: 2
Event: 15	Rank: 12	Min: 8	Max: 12	Conflict: 1
Event: 16	Rank: 21	Min: 18	Max: 21	Conflict: 1
Event: 17	Rank: 21	Min: 18	Max: 21	Conflict: 1
Event: 18	Rank: 21	Min: 18	Max: 21	Conflict: 1
Event: 19	Rank: 13.5	Min: 13.5	Max: 21	Conflict: 5
Event: 20	Rank: 21	Min: 13.5	Max: 21	Conflict: 1
Event: 21	Rank: 18	Min: 13.5	Max: 21	Conflict: 5
Event: 22	Rank: 13.5	Min: 13.5	Max: 18.5	Conflict: 2
Event: 23	Rank: 21	Min: 17	Max: 21	Conflict: 1
Event: 24	Rank: 26	Min: 26	Max: 26	Conflict: 0
Event: 25	Rank: 24	Min: 24	Max: 24	Conflict: 0
Event: 26	Rank: 25	Min: 25	Max: 25	Conflict: 0
Event: 27	Rank: 27	Min: 27	Max: 27	Conflict: 0

Node: 9 - Onychocaudata

Event: 1	Rank: 6.5	Min: 6.5	Max: 8.5	Conflict: 2
Event: 2	Rank: 4	Min: 2	Max: 8	Conflict: 5
Event: 3	Rank: 2	Min: 2	Max: 4	Conflict: 1
Event: 4	Rank: 2	Min: 2	Max: 4	Conflict: 1
Event: 5	Rank: 2	Min: 2	Max: 4	Conflict: 1
Event: 6	Rank: 10.5	Min: 5	Max: 10.5	Conflict: 4
Event: 7	Rank: 5	Min: 4	Max: 10.5	Conflict: 3
Event: 8	Rank: 8	Min: 4	Max: 12.5	Conflict: 6
Event: 9	Rank: 6.5	Min: 6.5	Max: 6.5	Conflict: 1
Event: 10	Rank: 9	Min: 7.5	Max: 10.5	Conflict: 3
Event: 11	Rank: 15.5	Min: 13.5	Max: 17	Conflict: 2
Event: 12	Rank: 15.5	Min: 13.5	Max: 17	Conflict: 2
Event: 13	Rank: 17	Min: 15.5	Max: 21	Conflict: 3
Event: 14	Rank: 10.5	Min: 9	Max: 10.5	Conflict: 2
Event: 15	Rank: 12	Min: 7.5	Max: 12	Conflict: 1
Event: 16	Rank: 21	Min: 18	Max: 21	Conflict: 1
Event: 17	Rank: 21	Min: 18	Max: 21	Conflict: 1
Event: 18	Rank: 21	Min: 18	Max: 21	Conflict: 1
Event: 19	Rank: 13.5	Min: 13.5	Max: 21	Conflict: 5
Event: 20	Rank: 21	Min: 13.5	Max: 21	Conflict: 1
Event: 21	Rank: 18	Min: 13.5	Max: 21	Conflict: 5
Event: 22	Rank: 13.5	Min: 13.5	Max: 18.5	Conflict: 2
Event: 23	Rank: 21	Min: 17	Max: 21	Conflict: 1
Event: 24	Rank: 26	Min: 26	Max: 26	Conflict: 0
Event: 25	Rank: 24	Min: 24	Max: 24	Conflict: 0
Event: 26	Rank: 25	Min: 25	Max: 25	Conflict: 0
Event: 27	Rank: 27	Min: 27	Max: 27	Conflict: 0

Node: 10 - Diplostraca

Event: 1	Rank: 7.5	Min: 7.5	Max: 7.5	Conflict: 1
Event: 2	Rank: 4	Min: 2	Max: 6	Conflict: 5
Event: 3	Rank: 2	Min: 2	Max: 6	Conflict: 2
Event: 4	Rank: 2	Min: 2	Max: 6	Conflict: 2
Event: 5	Rank: 2	Min: 2	Max: 6	Conflict: 2
Event: 6	Rank: 10.5	Min: 5	Max: 10.5	Conflict: 4
Event: 7	Rank: 5	Min: 4	Max: 10.5	Conflict: 3
Event: 8	Rank: 6	Min: 2	Max: 12.5	Conflict: 9
Event: 9	Rank: 7.5	Min: 5.5	Max: 7.5	Conflict: 2
Event: 10	Rank: 9	Min: 5.5	Max: 10.5	Conflict: 3
Event: 11	Rank: 15.5	Min: 15.5	Max: 17	Conflict: 1
Event: 12	Rank: 15.5	Min: 15.5	Max: 17	Conflict: 1
Event: 13	Rank: 17	Min: 15.5	Max: 23	Conflict: 3
Event: 14	Rank: 10.5	Min: 9	Max: 10.5	Conflict: 2
Event: 15	Rank: 12	Min: 5.5	Max: 12	Conflict: 1
Event: 16	Rank: 21	Min: 18	Max: 23	Conflict: 3
Event: 17	Rank: 21	Min: 18	Max: 23	Conflict: 3
Event: 18	Rank: 21	Min: 18	Max: 23	Conflict: 3
Event: 19	Rank: 13	Min: 13	Max: 19	Conflict: 2
Event: 20	Rank: 19	Min: 13	Max: 23	Conflict: 6
Event: 21	Rank: 18	Min: 13	Max: 21	Conflict: 6
Event: 22	Rank: 14	Min: 14	Max: 18.5	Conflict: 1
Event: 23	Rank: 23	Min: 17	Max: 26	Conflict: 6
Event: 24	Rank: 26	Min: 23	Max: 26	Conflict: 1
Event: 25	Rank: 24	Min: 24	Max: 24	Conflict: 0
Event: 26	Rank: 25	Min: 25	Max: 25	Conflict: 0
Event: 27	Rank: 27	Min: 27	Max: 27	Conflict: 0

Node: 11 - Phyllopoda

Event: 1	Rank: 7.5	Min: 7.5	Max: 7.5	Conflict: 1
Event: 2	Rank: 4	Min: 2	Max: 6	Conflict: 5
Event: 3	Rank: 2	Min: 2	Max: 6	Conflict: 2
Event: 4	Rank: 2	Min: 2	Max: 6	Conflict: 2
Event: 5	Rank: 2	Min: 2	Max: 6	Conflict: 2
Event: 6	Rank: 11	Min: 6	Max: 11	Conflict: 2
Event: 7	Rank: 5	Min: 4	Max: 6.5	Conflict: 2
Event: 8	Rank: 6	Min: 2	Max: 12.5	Conflict: 9
Event: 9	Rank: 7.5	Min: 5.5	Max: 7.5	Conflict: 2
Event: 10	Rank: 9	Min: 5.5	Max: 11	Conflict: 3
Event: 11	Rank: 15.5	Min: 15.5	Max: 18	Conflict: 2
Event: 12	Rank: 15.5	Min: 15.5	Max: 18	Conflict: 2
Event: 13	Rank: 17	Min: 15.5	Max: 20	Conflict: 4
Event: 14	Rank: 10	Min: 9	Max: 10	Conflict: 1
Event: 15	Rank: 12	Min: 5.5	Max: 12	Conflict: 1
Event: 16	Rank: 24	Min: 20	Max: 24	Conflict: 3
Event: 17	Rank: 24	Min: 20	Max: 24	Conflict: 3
Event: 18	Rank: 24	Min: 20	Max: 24	Conflict: 3
Event: 19	Rank: 13.5	Min: 13.5	Max: 22	Conflict: 3
Event: 20	Rank: 22	Min: 13.5	Max: 24	Conflict: 6
Event: 21	Rank: 21	Min: 13.5	Max: 24	Conflict: 6
Event: 22	Rank: 13.5	Min: 13.5	Max: 13.5	Conflict: 1
Event: 23	Rank: 20	Min: 17	Max: 27	Conflict: 9
Event: 24	Rank: 27	Min: 20	Max: 27	Conflict: 1
Event: 25	Rank: 19	Min: 19	Max: 20.5	Conflict: 1
Event: 26	Rank: 18	Min: 15.5	Max: 20.5	Conflict: 4
Event: 27	Rank: 26	Min: 26	Max: 26	Conflict: 0
