# Supplementary material for: Unraveling the origin of Cladocera by identifying heterochrony in the developmental sequences of Branchiopoda
Source: Front Zool. 2013 Jun 19;10:35. doi: 10.1186/1742-9994-10-35 (PMC3716531; doi:10.1186/1742-9994-10-35)
Supplement: Additional file 6 — Parsimov event-pairing analysis - DELTRAN Parsimov shifts. [file 1742-9994-10-35-S6.rtf]

Parsimov event-pairing analysis - DELTRAN Parsimov shifts


Summary of ASCAS-cracked data from "apodeltran_morph.txt"
Created by Parsimov 1.0.7g beta

For each node, a record of all the shortest
runs has been saved as a .out file.
These files have all have the suffix "-%655"

All characters used.  Exhaustive searches used if possible.
DelTran character-state optimization used

Original Tree:

                   /---------------------------------------------------------------------------------------- Anostraca
                   |
 -----------------11                /----------------------------------------------------------------------- Notostraca
                   |                |
                   \---------------10                 /----------------------------------------------------- Laevicaudata
                                    |                 |
                                    \-----------------9                 /----------------------------------- Spinicaudata
                                                      |                 |
                                                      \-----------------8                /------------------ Cyclestherida
                                                                        \----------------7
                                                                                         \------------------ Cladocera

======================================
Node 11 --> Anostraca
6 characters with informative movement
Thorough search using all possible unpolarised seed-combinations (720 iterations)

Consensus of 1 MS solution without conflicts
Consensus has 3/3 steps (100.0%)

Twins (6, 14)
Char 7 moved L relative to 1, 2
Char 19 moved E relative to 20, 21

======================================
Node 11 --> Node 10
11 characters with informative movement
Heuristic search using three unpolarised seeds (990 iterations)

Consensus of 6 MS solutions without conflicts
Consensus has 4/5 steps (80.0%)

Twins (6, 7) (24, 27)
Char 25 moved L relative to 16, 17, 18, 20
Char 26 moved L relative to 11, 12, 13, 16, 17, 18, 20, 25

======================================
Node 10 --> Notostraca
25 characters with informative movement
Heuristic search using three unpolarised seeds (13800 iterations)

Consensus of 67 MS solutions without conflicts
Consensus has 6/10 steps (60.0%)

Char 6 moved E relative to 1, 2, 3, 4, 5
Char 7 moved E relative to 3, 4, 5, 8
Char 19 moved L relative to 16, 17, 18
Char 22 moved L relative to 16, 17, 18, 20
Char 23 moved L relative to 16, 17, 18, 20
Char 24 moved E relative to 25, 26

======================================
Node 10 --> Node 9
7 characters with informative movement
Thorough search using all possible unpolarised seed-combinations (5040 iterations)

Consensus of 1 MS solution without conflicts
Consensus has 4/4 steps (100.0%)

Twins (21, 20) (23, 24)
Char 8 moved L relative to 3, 4, 5
Char 19 moved L relative to 11, 12

======================================
Node 9 --> Laevicaudata
22 characters with informative movement
Heuristic search using three unpolarised seeds (9240 iterations)

Consensus of 42 MS solutions without conflicts
Consensus has 9/9 steps (100.0%)

Twins (24, 26)
Char 8 moved L relative to 1, 2, 6, 11, 12, 13, 16, 17, 18, 19, 20, 22, 23
Char 11 moved L relative to 16, 17, 18, 20, 23
Char 12 moved L relative to 16, 17, 18, 20, 23
Char 13 moved L relative to 16, 17, 18, 20
Char 14 moved E relative to 1, 2, 3, 4, 5, 9, 10
Char 15 moved E relative to 6, 10
Char 19 moved L relative to 13, 16, 17, 18, 22, 23
Char 21 moved E relative to 6, 10, 15, 16, 17, 18

======================================
Node 9 --> Node 8
0 characters with informative movement
No nontrivial seeds (0 iterations)

Consensus of 1 MS solution without conflicts
Consensus has 1/1 steps (100.0%)

Twins (10, 7)

======================================
Node 8 --> Spinicaudata
22 characters with informative movement
Heuristic search using three unpolarised seeds (9240 iterations)

Consensus of 200 MS solutions without conflicts
Consensus has 10/12 steps (83.3%)

Twins (27, 24)
Char 6 moved L relative to 8, 10, 11, 12
Char 7 moved L relative to 1, 2, 9, 11, 12, 14
Char 8 moved L relative to 1, 2, 14
Char 10 moved L relative to 8
Char 13 moved L relative to 11, 12, 16, 17, 18, 20
Char 21 moved E relative to 11, 12, 16, 17, 18
Char 22 moved E relative to 19, 21
Char 25 moved E relative to 16, 17, 18, 23
Char 26 moved E relative to 16, 17, 18, 23

======================================
Node 8 --> Node 7
24 characters with informative movement
Heuristic search using three unpolarised seeds (12144 iterations)

Consensus of 692 MS solutions without conflicts
Consensus has 10/12 steps (83.3%)

Char 3 moved L relative to 1, 2, 7, 8, 9, 11, 12, 17
Char 4 moved L relative to 1, 2, 7, 8, 9, 11, 12, 17
Char 6 moved E relative to 1, 2, 7, 14
Char 9 moved L relative to 8, 11, 12
Char 10 moved E relative to 1, 2, 14
Char 13 moved E relative to 1, 2, 7, 11, 12, 14, 19
Char 15 moved L relative to 8, 11, 12, 17
Char 19 moved E relative to 14, 21
Char 20 moved E relative to 14, 16, 18, 21
Char 22 moved L relative to 11, 12, 16, 17, 18

======================================
Node 7 --> Cyclestherida
18 characters with informative movement
Heuristic search using three unpolarised seeds (4896 iterations)

Consensus of 128 MS solutions without conflicts
Consensus has 1/10 steps (10.0%)

Char 17 moved E relative to 16, 18

======================================
Node 7 --> Cladocera
20 characters with informative movement
Heuristic search using three unpolarised seeds (6840 iterations)

Consensus of 79 MS solutions without conflicts
Consensus has 9/9 steps (100.0%)

Char 3 moved L relative to 14, 16, 18, 19, 21
Char 4 moved L relative to 14, 16, 18, 19, 21
Char 8 moved L relative to 6, 7, 13
Char 9 moved L relative to 14, 19
Char 14 moved L relative to 11, 12
Char 15 moved L relative to 16, 18, 21
Char 20 moved E relative to 11, 12, 17
Char 24 moved E relative to 16, 17, 18, 21, 22
Char 26 moved E relative to 16, 17, 18, 21, 22

======================================

Run took 0d, 0h, 6m, 1s
